# Reprogramming of cancer metabolism via photoresponsive nano-PROTAC enhances pyroptosis-mediated immunotherapy

**DOI:** 10.1038/s41392-025-02405-6

**Published:** 2025-09-26

**Authors:** Byeongmin Park, Jiwoong Choi, Jae-Hyeon Lee, Yelee Kim, Woohyeong Lee, Ansoo Lee, In-Cheol Sun, Hong Yeol Yoon, Yongju Kim, Sun Hwa Kim, Yoosoo Yang, Kwangmeyung Kim, Jooho Park, Man Kyu Shim

**Affiliations:** 1 Biomedical Research Division, Medicinal Materials Research Center, Korea Institute of Science and Technology (KIST), Seoul, Republic of Korea; 2 KU-KIST Graduate School of Converging Science and Technology, Korea University, Seoul, Republic of Korea; 3 BK21 Program, Department of Applied Life Science, Konkuk University, Chungju, Republic of Korea; 4 Division of Bio-Medical Science and Technology, KIST School, University of Science and Technology, Seoul, Republic of Korea; 5 Department of Integrative Biotechnology, Sungkyunkwan University, Suwon, Republic of Korea; 6 College of Pharmacy, Graduate School of Pharmaceutical Sciences, Ewha Womans University, Seoul, Republic of Korea; 7 Gradutate Program in Innovative Biomaterials Convergence, Ewha Womans University, Seoul, Republic of Korea; 8 Present Address: Department of Immunology, School of Medicine, Kyungpook National University, Daegu, 41944 Republic of Korea

**Keywords:** Drug delivery, Drug development

## Abstract

Photodynamic therapy (PDT) induces tumor cell pyroptosis, a form of programmed cell death that triggers antitumor immunity. However, high glucose metabolism and hypoxic conditions in the tumor microenvironment (TME) limit PDT efficiency and impair effector cell function. Here, we propose a cancer metabolic reprogramming-enabling photoresponsive nanoproteolysis-targeting chimera (Nano-PROTAC; NanoTAC), derived from the supramolecular self-assembly of drug conjugates that bridge a PROTAC targeting hexokinase II (HK2) and a photosensitizer via a biomarker-cleavable linker. In a triple-negative breast cancer (TNBC) model, NanoTAC initially silences PROTAC activity and accumulates in tumor regions, where it undergoes linker cleavage in response to enzymatic biomarkers. Upon photoirradiation, PDT-induced pyroptotic cell death promotes the release of tumor-associated antigens (TAAs) and damage-associated molecular patterns (DAMPs) to drive the cancer-immunity cycle. Concurrently, targeted protein degradation (TPD) via PROTACs counteracts glucose and oxygen consumption in the TME, ultimately potentiating pyroptosis-mediated photoimmunotherapy. This combination therapy achieves a high rate of complete regression in primary TNBC and confers adaptive immunity to prevent metastasis and recurrence. Our study presents a rationally designed nanomedicine that integrates PDT and PROTACs, shedding light on strategies for more effective cancer immunotherapy.

## Introduction

Pyroptosis, a highly inflammatory form of programmed cell death, is initiated by the cleavage of gasdermin D (GSDMD) following caspase-1 activation under specific conditions encountered by tumor cells.^1–3^ This process induces distinctive morphological changes, such as cellular swelling and lysis with large bubbles driven by pore formation in the cell membrane, resulting in the release of danger signals and inflammatory cytokines.^4–6^ Compared with apoptosis, pyroptotic cell death is increasingly recognized as a more effective pathway for cancer immunotherapy,^7–9^ as it drives the cancer-immunity cycle by recruiting effector cells into the tumor microenvironment (TME), converting cold tumors into hot tumors, and markedly enhancing both innate and adaptive immune responses.^10–12^ Numerous pyroptosis agents, including ions, small-molecule drugs, and gene therapeutics, have been reported in recent literature, highlighting their promising potential to activate pyroptotic pathways across diverse cancer types.^13^ However, given that off-target pyroptosis inevitably provokes severe damage to normal tissue due to its inflammatory nature, strategies that can selectively induce pyroptotic tumor cell death to improve both the efficacy and safety of immunotherapy are urgently needed.^14^

Photodynamic therapy (PDT) has garnered considerable clinical interest for the treatment of intractable solid tumors, offering key advantages such as minimal invasiveness and exceptionally high spatiotemporal precision.^15–19^ During the photodynamic process, reactive oxygen species (ROS) generated by photosensitizers at tumor sites locally exposed to visible light activate the inflammasome and induce pyroptotic cell death.^20,21^ Hence, PDT holds significant promise for immunotherapy because it enables precisely controlled tumor pyroptosis, but the limited targeting efficiency and poor retention of photosensitizers in tumor tissues have restricted its broader application.^22^ The hypoxic TME presents an additional barrier that compromises the therapeutic efficacy of PDT, which relies heavily on oxygen availability.^23,24^ Under these hypoxic conditions, elevated glycolysis becomes a metabolic hallmark of tumor cells, leading to a TME that impairs the infiltration of effector cells by depleting nutrients and enriching immunosuppressive metabolites.^25,26^ Even in cancer cells that depend on Warburg-type metabolism, unilateral inhibition of glycolysis often increases the oxygen consumption rate (OCR) due to compensatory upregulation of mitochondrial oxidative phosphorylation (OXPHOS).^27^ Therefore, coordinated regulation of both the glycolytic pathway and mitochondrial respiration is necessary to offset oxygen consumption in cancer cells, which can enhance the efficacy of PDT-mediated immunotherapy.

Leveraging advances in nanotechnology, various strategies have been developed to deliver glycolytic inhibitors for metabolic reprogramming in the TME together with photosensitizers, thereby enhancing the overall therapeutic outcomes of PDT.^28–34^ Nevertheless, conventional nanoparticulate drug delivery systems based on polymers, proteins, lipids, and other organic/inorganic substances (e.g., metal oxides, carbon, silica, or metal–organic framework) intrinsically suffer from low payload capacities (typically less than 10%) because of the requirement for excessive carrier materials in drug formulations.^20,35^ These carriers therefore inherently pose toxic risks associated with immunogenicity, and their noncovalent cargo encapsulation often results in premature and uncontrolled drug leakage at off-target sites.^36,37^ From an industrial standpoint, the complex and heterogeneous architecture of traditional nanomedicines complicates synthetic optimization, mass production, and quality control (QC), all of which continue to hinder clinical translation.^38^ A recently emerged major challenge in translating nanoparticles to the clinic is their unexpectedly low delivery efficiency, with fewer than 1% of administered doses reaching tumor tissues.^39^ This fundamental limitation affects manufacturing, drug cost, therapeutic efficacy, and biosafety, underscoring the need for more straightforward and reproducible formulation technologies.

**Scheme 1:**
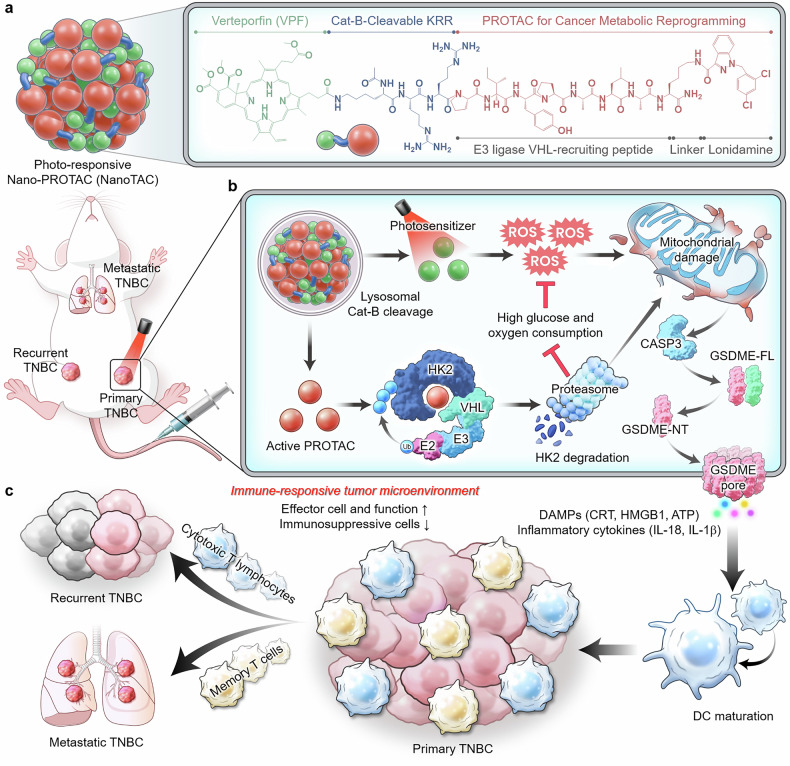
Design of NanoTAC and mechanism of cancer metabolic reprogramming to enhance PDT-mediated pyroptosis. **a** Chemical structure of the drug conjugate comprising NanoTAC. NanoTAC self-assembles spontaneously from drug conjugates that include an HK2-targeting PROTAC (red), a cathepsin B-cleavable peptide KRR (blue), and the photosensitizer verteporfin (VPF; green) without requiring any additional carrier materials. **b** Process of NanoTAC-mediated photoimmunotherapy. In a TNBC mouse model, NanoTAC accumulates in tumors and becomes enzymatically activated by lysosomal cathepsin B, inducing pyroptosis via mitochondrial damage upon laser irradiation. This leads to GSDME pore formation, as activated caspase-3 (CASP3) cleaves GSDME-FL into GSDME-NT fragments, resulting in the extracellular release of DAMPs and inflammatory cytokines. Moreover, PROTAC-mediated TPD eliminates intracellular HK2, which independently damages mitochondria and counteracts glucose and oxygen consumption during the photodynamic process. **c** This synergistic combination of PDT and PROTAC triggers significantly amplified pyroptotic tumor cell death, drives the cancer-immunity cycle to recruit effector cells, and ultimately inhibits primary, metastatic, and recurrent TNBC

In this work, we report a photoresponsive nanoproteolysis-targeting chimera (Nano-PROTAC; NanoTAC) that self-assembles supramolecularly from drug conjugates consisting of a PROTAC and a photosensitizer connected via an enzymatic biomarker-cleavable linker (Scheme 1a). By eliminating the dependence on carrier materials, this small-molecule prodrug structure, which is suitable for large-scale production and quality control, spontaneously organizes into nanoparticles through intermolecular interactions, achieving high drug loading and enabling selective drug release mediated by the target biomarker.^40^ In a triple-negative breast cancer (TNBC) mouse model, intravenously administered NanoTAC accumulates in tumor lesions due to the enhanced permeability and retention (EPR) effect and undergoes enzymatic activation by the cancer biomarker cathepsin B (Cat-B).^41^ Upon laser irradiation, the ROS generated by PDT induce pyroptotic cell death, triggering the release of tumor-associated antigens (TAAs) and damage-associated molecular patterns (DAMPs), which promote dendritic cell (DC) maturation and T-cell cross-priming (Scheme 1b).^42^ Simultaneously, PROTAC-mediated targeted protein degradation (TPD) of hexokinase II (HK2) suppresses both glycolysis and mitochondrial respiration, thereby counteracting oxygen consumption in the TME during the photodynamic process.^43^ We show that this therapeutic strategy, which combines PDT and cancer metabolic reprogramming via PROTACs within a rationally coordinated nanomedicine, leads to a pathological complete response in TNBC and establishes adaptive immunity that inhibits metastasis and recurrence (Scheme 1c). These findings underscore the therapeutic potential of PROTAC-assisted PDT by NanoTAC in refractory cancers, offering insights for the development of advanced immunotherapeutic strategies. As a generalizable platform also applicable to other target proteins, our study highlights the possibility to expand PROTAC applications in cancer therapy.

## Results

### Design, preparation, and characterization

NanoTAC is derived from the supramolecular self-assembly of drug conjugates composed of purposefully tailored components, including (i) a PROTAC constructed from the HK2-selective inhibitor lonidamine,^44,45^ a lysine residue serving as a four-atom linker^46^ and the E3 ligase VHL-recruiting peptide ALAPYIP;^47–49^ (ii) KRR, a substrate peptide for the cancer biomarker cathepsin B;^50^ and (iii) the clinically approved photosensitizer verteporfin (VPF), marketed under the trade name Visudyne^®^.^51^ As shown in Supplementary Figs. 1–6, the drug conjugate was synthesized through a straightforward three-step chemical reaction, in which the final product and all intermediates were characterized by LC–MS and proton nuclear magnetic resonance (^1^H-NMR).

**Fig. 1:**
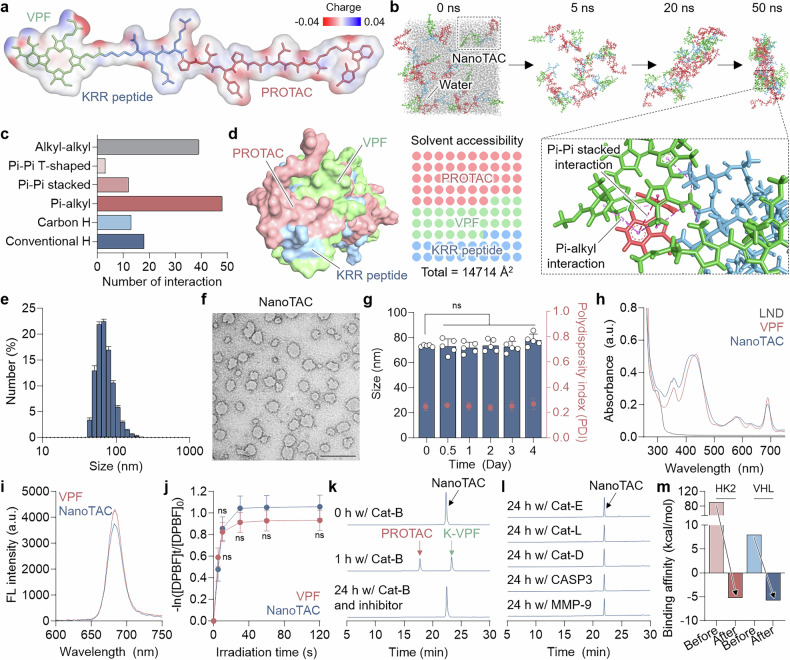
Physicochemical Characterization of NanoTAC. **a** Electrostatic potential surface and schematic representation of the drug conjugate comprising NanoTAC. **b** Representative MD simulation snapshots showing the time-dependent self-assembly behavior of the drug conjugates in an explicit water box. **c** Classification of major noncovalent interactions observed at 50 ns of simulation time. **d** Molecular surface model of NanoTAC and solvent accessibility mapping of individual molecular domains, presented by component and total surface areas. **e** Size distribution profiles of NanoTAC. **f** TEM image of NanoTAC. Scale bar, 500 nm. **g** Particle stability of NanoTAC in mouse serum. Changes in particle size and polydispersity index over time were analyzed using dynamic light scattering (DLS; n = 5). **h** UV‒vis absorbance and **i** fluorescence (FL) spectra of the indicated compounds. **j** ROS generation by NanoTAC and VPF under laser irradiation at a power of 40 mW (n = 5). Cleavage behavior of NanoTAC after incubation in **k** Cat-B alone or in combination with Z-FA-FMK or **l** other enzymes, including Cat-E, Cat-L, Cat-D, CASP3, and MMP-9. **m** Changes in the binding affinity of NanoTAC for HK2 or VHL before and after cleavage. Statistical significance was determined by Student’s t-test (**j**) and one-way ANOVA with Tukey‒Kramer post hoc test (**g**)

We first investigated the driving force behind the supramolecular assembly of the drug conjugates via molecular dynamics (MD) simulations. Their amphiphilic nature was identified by generating an electrostatic potential map (Fig. 1a). Drug-conjugating molecules randomly placed in a cubic water box were gradually compacted into a single cluster (Fig. 1b). This aggregation process correlated with a gradual increase in van der Waals forces, hydrogen bonding and hydrophobic interactions, indicating that spontaneous self-assembly was accompanied by progressive structural stabilization over time (Supplementary Fig. 7). Specifically, various hydrophobic interactions—including 39 alkyl–alkyl, 12 π–π stacking and 48 π–alkyl interactions between the VPF and LND moieties of the drug conjugates—played a critical role in stabilizing the assembled structure (Fig. 1c). Further insights were gained through solvent accessibility analysis, which reflects the exposure of molecular components to solvents; lower accessibility suggests greater hydrophobicity and potential core formation within the nanostructures.^52^ The total solvent-accessible surface area (SASA) of NanoTAC, derived from the supramolecular self-assembly of the drug conjugates, was calculated as 14,714 Å^2^ (Fig. 1d). The PROTAC moiety accounted for the majority of surface exposure, potentially contributing to nanoparticle stability, whereas the VPF and KRR peptides exhibited relatively limited surface accessibility. The minimal surface exposure of the cleavable linker may contribute to preventing nonspecific degradation.

The self-assembled NanoTAC exhibited a uniform size distribution, with a hydrodynamic diameter of 73.58 ± 0.72 nm and a spherical morphology (Fig. 1e, f). The zeta potential was determined to be 13.14 ± 1.59 mV. Furthermore, no significant changes in average size or polydispersity index were observed over 4 days in mouse serum, indicating exceptional stability under physiological conditions (Fig. 1g and Supplementary Fig. 8). UV‒vis absorption spectra of NanoTAC revealed two characteristic peaks corresponding to lonidamine and VPF, and its fluorescence emission profile was similar to that of VPF, with a peak at 670 nm (Fig. 1h, i and Supplementary Fig. 9). Their photodynamic properties were assessed via 1,3-diphenylisobenzofuran (DPBF) analysis, which revealed that the generation of ROS, with a quantum yield (Φ) ≈ 0.7823, was comparable to that of VPF (Φ = 0.78) under laser irradiation (Fig. 1j). These results demonstrate that the chemical modification of VPF into drug conjugates does not compromise its photoactivity.

Target enzyme-specific activation was evaluated by incubating NanoTAC with various cancer-associated biomarkers. Rapid conversion into PROTAC and lysine-conjugated VPF (K-VPF) was observed within 1 h of incubation with Cat-B, accompanied by a concentration-dependent increase in cleavage kinetics; the catalytic efficiency (*k*_cat_/*K*_M_) was determined to be 27,898 M^−1^S^−1^ (Fig. 1k and Supplementary Fig. 10). In contrast, no detectable cleavage occurred in the presence of Cat-B along with an irreversible cysteine protease inhibitor. NanoTAC also exhibited no cleavage behavior when incubated with other enzymes, such as Cat-E, Cat-L, Cat-D, caspase-3 (CASP3), or matrix metalloproteinase-9 (MMP-9; Fig. 1l). Therefore, NanoTAC can undergo selective enzymatic cleavage to release the active PROTAC and photosensitizer in tumor cells, potentially reducing adverse effects from off-target HK2 proteolysis.^53^ This notion is supported by molecular binding simulations, which revealed that intact NanoTAC fails to bind either to target HK2 or VHL but exhibits meaningful binding affinities after cleavage into PROTACs (Fig. 1m, Supplementary Figs. 11 and 12). Biolayer interferometry (BLI) analysis further confirmed that the PROTAC cleaved from NanoTAC binds to HK2 and VHL, with K_D_ values of 3.48 nM and 5.29 nM, respectively (Supplementary Fig. 13).

### In vitro glycolysis inhibition and photodynamic activity

**Fig. 2:**
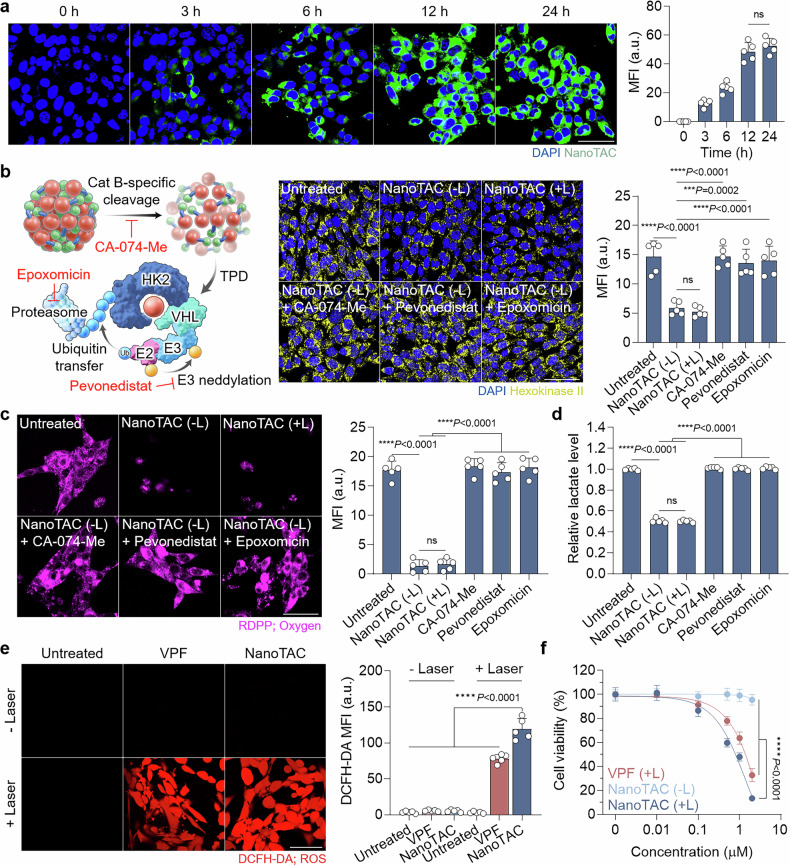
In vitro glycolysis inhibition and photodynamic activity of NanoTAC. **a** Representative CLSM images of 4T1 cells incubated with NanoTAC. MFI data are presented as the mean ± SD (n = 5). Scale bar, 50 μm. **b** Schematic illustration of the mechanism underlying TPD by NanoTAC and the related inhibitors, along with representative CLSM images of HK2-stained 4T1 cells after the indicated treatments. MFI data are presented as the mean ± SD (n = 5). Scale bar, 50 μm. **c** Oxygen and **d** extracellular lactate levels in 4T1 cells after the indicated treatments (n = 5). MFI data are presented as the mean ± SD. Scale bar, 50 μm. **e** ROS generation in 4T1 cells following treatment with NanoTAC or VPF in the absence or presence of laser irradiation (40 mW, 500 s). MFI data are presented as the mean ± SD (n = 5). Scale bar, 50 μm. **f** Viability of 4T1 cells treated with varying concentrations of NanoTAC or VPF in the absence or presence of laser irradiation (40 mW, 500 s; n = 5). Statistical significance was determined by one-way ANOVA with Tukey‒Kramer post hoc test (**a**–**f**)

By tracking the distinct fluorescence signals of VPF, we examined the cellular uptake of NanoTAC in the murine mammary carcinoma cell line 4T1, which mimics stage IV TNBC and overexpresses Cat-B.^54–56^ Its cellular accumulation increased gradually in a time-dependent manner, plateauing after 12 h of treatment, similar to VPF (Fig. 2a and Supplementary Fig. 14). We then assessed changes in the target protein HK2, which is associated with glycolytic pathways in tumor cells. Treatment with NanoTAC significantly decreased intracellular HK2 levels, whereas no target protein degradation occurred in 4T1 cells pretreated with inhibitors of key steps in the TPD process of NanoTAC, including CA-074-Me (a cathepsin B inhibitor), pevonedistat (a NEDD8-activating enzyme inhibitor), and epoxomicin (a proteasome inhibitor; Fig. 2b and Supplementary Fig. 15).^57^ These findings indicate that NanoTAC induces a proteolytic process involving HK2 and VHL binding and the proteasome-ubiquitination cascade (i.e., HK2 neddylation) following enzymatic activation by the target biomarker. Notably, HK2 expression remained suppressed in NanoTAC-treated cells after laser irradiation (+L), suggesting the potential for sustained TPD during PDT. Notably, treatment with lonidamine led to an initial reduction in the intracellular HK2 level followed by eventual regeneration, whereas NanoTAC induced sustained degradation of the target protein in TNBC cells through the recyclable action of PROTACs (Supplementary Fig. 16). Consistent with these results, oxygen consumption was minimized in 4T1 tumor cells treated with NanoTAC (−L or +L), as indicated by the quenched fluorescence signals of [Ru(dpp)_3_]Cl_2_ (RDPP) due to sufficient intracellular oxygen compared with other conditions (Fig. 2c).^58,59^ These cells also presented reduced levels of lactate, a well-established end product of glycolysis (Fig. 2d).^60^ Although glycolytic inhibition typically increases cellular oxygen consumption by increasing mitochondrial oxidative phosphorylation as a compensatory mechanism, LND reduces it by concurrently suppressing both glycolysis and mitochondrial respiration.

To elucidate the enhanced PDT efficiency resulting from HK2 proteolysis, we evaluated ROS generation and cytotoxicity under culture conditions. Under laser irradiation (+L), 4T1 cells treated with NanoTAC presented significantly greater intracellular ROS generation than did those treated with VPF (Fig. 2e). Considering that type II PSs utilize molecular oxygen to generate ROS, the increased ROS production relative to that of VPF (+L) is attributed to the utilization of sufficient oxygen availability in NanoTAC (+L)-treated cells.^61^ Inhibiting glycolysis via HK2 degradation by NanoTAC in the absence of photoirradiation (−L) led to minimal TNBC cell death (Fig. 2f). In contrast, NanoTAC (+L) induced substantially greater cytotoxicity than both NanoTAC (−L) and VPF (+L). We also confirmed that NanoTAC effectively counteracts the accelerated oxygen consumption observed in untreated or VPF-treated cells under hypoxic conditions that mimic the in vivo TME, thereby enabling sufficient ROS generation upon photoirradiation (Supplementary Fig. 17). Compared with that under normoxia, ROS production by NanoTAC (+L) in tumor cells under hypoxia was reduced by 36.1%, whereas VPF (+L) showed a more pronounced reduction of 79.1%. These results highlight the superior PDT efficacy achieved by eliminating metabolic barriers in tumors through PROTAC activity.

### Enhanced pyroptosis to increase antitumor immunity

**Fig. 3:**
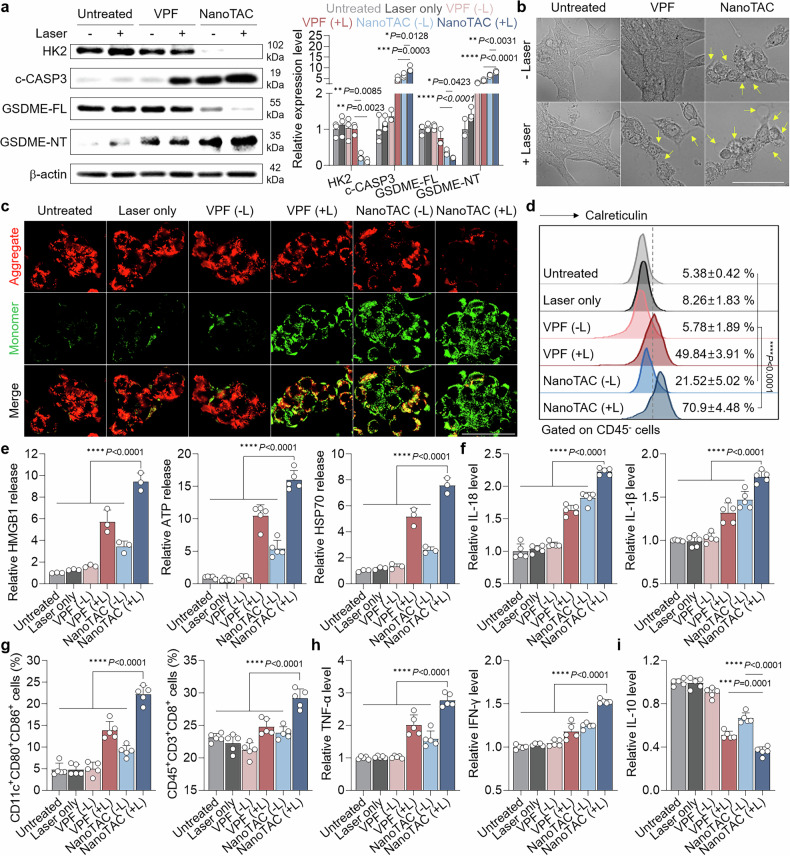
Enhanced pyroptosis to boost antitumor immunity. **a** Pyroptosis-related protein expression and **b** morphology of 4T1 cells treated with NanoTAC or VPF in the absence or presence of laser irradiation (40 mW, 500 s). Quantitative data are presented as the mean ± SD (n = 3). Scale bar, 100 μm. **c** Mitochondrial membrane potential (Δψm) visualized by JC-1 dye in 4T1 cells following the indicated treatments. Scale bar, 50 μm. **d** Surface CRT expression and **e**, **f** extracellular release of HMGB1, ATP, HSP70, IL-18, and IL-1β in 4T1 cells following the indicated treatments (n = 5). **g** Populations of mature DCs and CTLs in splenocytes after coculture with 4T1 cells treated with NanoTAC or VPF in the absence or presence of laser irradiation (40 mW, 500 s; n = 5). Levels of **h** TNF-α, IFN-γ, and **i** IL-10 in the medium after coculture of splenocytes with 4T1 cells following the indicated treatments (n = 5). Statistical significance was determined by one-way ANOVA with Tukey‒Kramer post hoc test (**a**, **d**–**i**)

To verify the ability of NanoTAC to promote pyroptosis, we monitored changes in relevant protein markers and cell morphology. Gasdermin E (GSDME) and CASP3 are key executors of pyroptotic pathways.^62,63^ TNBC cells treated with NanoTAC (+L) underwent GSDME cleavage, as evidenced by a reduction in full-length GSDME (GSDME-FL) and an increase in GSDME-N-terminal (GSDME-NT) fragments generated by upregulated cleaved CASP3 (c-CASP3; Fig. 3a). These cells also displayed swelling and large bubbles on the plasma membrane, the hallmarks of pyroptotic cell death (Fig. 3b).^20^ The Δψm was assessed by JC-1 dye, which differentiates between intact and damaged mitochondria by red and green fluorescence corresponding to high and low Δψm, respectively.^64^ As shown in Fig. 3c, intense green fluorescence was observed in NanoTAC (+L)-treated 4T1 cells, indicating that pyroptotic death was initiated by mitochondrial damage. The NanoTAC (−L) group exhibited perceptible pyroptotic features, as HK2 degradation independently triggered mitochondrial disruption. Cellular HK2 suppresses the mitochondrial translocation of the proapoptotic proteins BAD and BAX and inhibits CASP3 activation.^65^ Recently, HK2 degradation has been reported to cause mitochondrial damage, which in turn triggers CASP3 cleavage and subsequent GSDME-dependent pyroptotic tumor cell death.^66^ Importantly, all these physicochemical alterations in tumor cells were more pronounced following NanoTAC (+L) treatment than in the NanoTAC (−L) or VPF (+L) groups. This enhanced effect arises from PROTAC activity, which suppresses glycolysis and damages mitochondria, acting synergistically with PDT to induce extensive pyroptosis and amplify the immunogenicity of dying cells.^67^

Robust pyroptotic tumor cell death in the NanoTAC (+L) group elicited substantial expression of DAMPs, as indicated by prominent calreticulin (CRT) translocation to the cell surface and extracellular release of soluble mediators such as high mobility group box 1 (HMGB1), adenosine triphosphate (ATP) and heat shock protein 70 (HSP70; Fig. 3d, e and Supplementary Fig. 18).^10^ These cells also release proinflammatory cytokines through the membrane pores formed by GSDME-NT.^68^ The extracellular release of interleukin-18 (IL-18) and IL-1β from TNBC cells markedly increased following NanoTAC (+L) treatment relative to all other conditions (Fig. 3f). Encouraged by the abundant release of immunogenic substances, we evaluated antitumor immunity in a coculture system. 4T1 cells treated with NanoTAC (+L) significantly increased the proportions of mature DCs (CD11c^+^CD80^+^CD86^+^) and cytotoxic T lymphocytes (CTLs; CD45^+^CD3^+^CD8^+^) among cocultured lymphocytes compared with those in the other groups (Fig. 3g and Supplementary Fig. 19). Moreover, the levels of tumor necrosis factor-alpha (TNF-α) and interferon-gamma (IFN-γ) released from activated CTLs into the coculture medium were elevated in the NanoTAC (+L) group and were 1.38- and 1.75-fold and 1.3- and 1.23-fold greater than those in the NanoTAC (−L) and VPF (+L) groups, respectively (Fig. 3h). In contrast, such extracellular release of the anti-inflammatory cytokine IL-10 decreased in cocultures containing tumor cells treated with NanoTAC (+L; Fig. 3i). These findings imply that NanoTAC-mediated pyroptosis promotes the cancer-immunity cycle, leading to an immune-hot TME.^69^

### Biodistribution and antitumor efficacy in an orthotopic TNBC mouse model

**Fig. 4:**
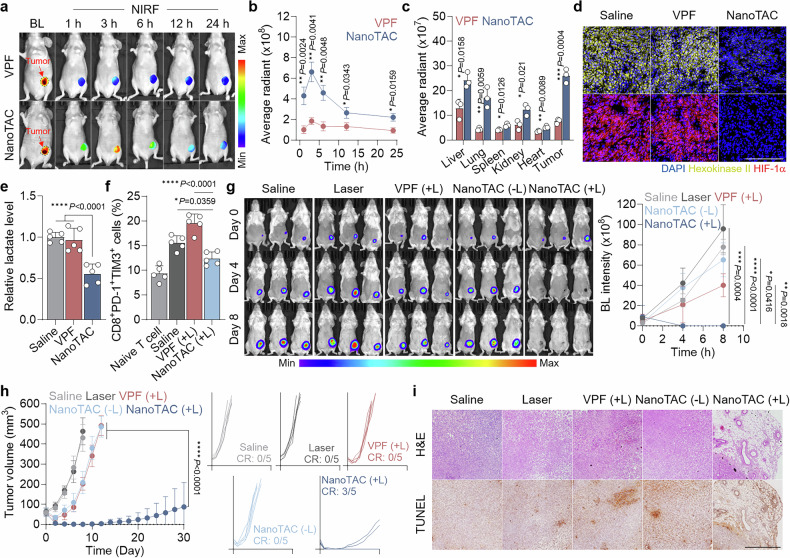
Biodistribution and antitumor efficacy of NanoTAC. **a** BL and tumor-mapping NIRF images of orthotopic TNBC models after intravenous administration of VPF or NanoTAC. **b** Quantitative fluorescence intensity in the tumor region presented as the mean ± SD (n = 3). **c** Fluorescence intensity of intravenously administered VPF or NanoTAC in major organs and tumor tissue (n = 3). **d** Representative CLSM images of tumor tissues stained for HK2 or HIF-1α. Scale bar, 100 μm. **e** Lactate levels in tumor supernatants (n = 5). **f** Populations of T cells expressing PD-1 and TIM3 following incubation in tumor supernatants from TNBC mice treated with saline, VPF (+L), or NanoTAC (+L; n = 5). **g** Representative time-course BL images of orthotopic TNBC mice following treatment with saline, laser only (Laser), VPF (+L), or NanoTAC (−L or +L). Quantitative luminescence intensity in the tumor region is presented as the mean ± SD (n = 5). **h** TNBC growth curves in each group (n = 5). **i** Representative images of tumor tissues stained with H&E or TUNEL. Scale bar, 500 μm. Statistical significance was determined by Student’s t-test (**b**, **c**) and one-way ANOVA with Tukey‒Kramer post hoc test (**e**–**h**)

To determine the optimal timing for laser irradiation, biodistribution and tumor accumulation kinetics were investigated via real-time near-infrared fluorescence (NIRF) imaging. Following intravenous administration in an orthotopic TNBC mouse model, NanoTAC accumulated in the tumor region, as indicated by bioluminescence (BL), during the initial 3 h, followed by time-course elimination (Fig. 4a). VPF displayed a comparable biodistribution pattern but relatively limited tumor targeting due to its small-molecule nature (Fig. 4b). Ex vivo NIRF imaging further confirmed 3.57-fold greater tumor accumulation of NanoTAC than VPF (Fig. 4c and Supplementary Fig. 20). As shown in Supplementary Fig. 21, NanoTAC exhibited significantly prolonged in vivo circulation, with detectable amounts remaining in the blood for up to 48 h and displaying enhanced pharmacokinetic (PK) parameters compared with those of VPF. Therefore, its tumor-targeting ability is attributed to the passive accumulation of long-circulating nanosized particles via the EPR effect.^70^

Systemic administration of NanoTAC promoted a TME with sufficient oxygen availability to support efficient PDT by inhibiting glycolysis and mitochondrial respiration through tumor HK2 degradation, resulting in depletion of hypoxia-inducible factor-1 alpha (HIF-1α) and extracellular lactate compared with the basal levels in the saline group (Fig. 4d, e and Supplementary Fig. 22). As a result, tumor tissues exposed to photoirradiation following NanoTAC treatment generated greater amounts of ROS than did those treated with VPF (Supplementary Fig. 23). In addition, the reduced level of the immunosuppressive metabolite lactate in the TME prevented the upregulation of exhaustion markers observed in T cells after incubation with tumor supernatants from the other groups (Fig. 4f and Supplementary Fig. 24). These findings verify that NanoTAC not only directly enhances PDT efficiency by suppressing oxygen consumption but also alleviates immune cell dysfunction, thereby further amplifying the effects of photoimmunotherapy.

The therapeutic efficacy of NanoTAC-mediated photoimmunotherapy was evaluated across five groups: (i) saline, (ii) laser only (laser), (iii) VPF (+L), (iv) NanoTAC (−L), and (v) NanoTAC (+L). BL imaging of orthotopic TNBC mice allowed direct visualization of tumor progression, revealing significantly lower BL intensity in the tumor region of the NanoTAC (+L) group (Fig. 4g). Notably, a proportion (60%) of the mice treated with NanoTAC (+L) experienced complete regression (CR) within 4 days post-treatment, in striking contrast to the other groups, which presented no CR (Fig. 4h). Histological analyses revealed substantial tissue damage along with apoptotic cell death in tumors following treatment with NanoTAC (+L, Fig. 4i). In agreement with these results, the mice in the NanoTAC (+L) group exhibited significantly prolonged survival (Supplementary Fig. 25). No noticeable body weight loss occurred in any of the groups throughout the treatment period (Supplementary Fig. 26). Ex vivo NIRF imaging (Fig. 4c) indicated that NanoTAC was partially retained in major organs after administration, yet no damage was observed in normal tissues (Supplementary Fig. 27). This in vivo safety arises from NanoTAC’s exceptional selectivity for its target enzyme, allowing enzymatic activation exclusively in TNBC cells while resisting cleavage by other endogenous enzymes or low cathepsin B levels at off-target sites, thereby preventing toxic side effects associated with nonspecific HK2 degradation (Supplementary Fig. 28).^43^

### Innate and adaptive immune responses in primary and recurrent TNBC

**Fig. 5:**
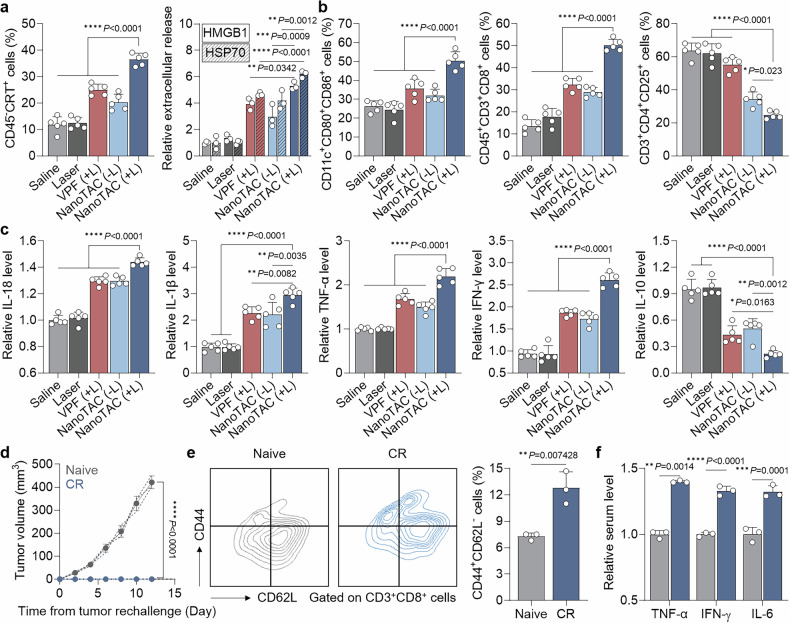
Innate and adaptive immune responses by NanoTAC in primary and recurrent TNBC. **a** Intratumoral CRT-positive tumor cells and extracellular release of HMGB1 and HSP70 in orthotopic TNBC mice following treatment with saline, laser only (Laser), VPF (+L), or NanoTAC (−L or +L; n = 5). **b** Populations of mature DCs, CTLs, and regulatory T cells in tumor tissues (n = 5). **c** Quantities of IL-18, IL-1β, TNF-α, IFN-γ and IL-10 in tumor supernatants (n = 5). **d** Growth curves of rechallenged tumors in CR and naive mice. Dashed lines indicate individual tumor growth curves in each group (n = 3). **e** Population of splenic T_em_ cells and **f** serum levels of TNF-α, IFN-γ, and IL-6 on day 15 after tumor rechallenge (n = 3). Statistical significance was determined by Student’s t-test (**d**–**f**) and one-way ANOVA with Tukey‒Kramer post hoc test (**a**–**c**)

Next, we examined DAMPs, immune cell populations and cytokine profiles in tumor tissues from orthotopic TNBC mice treated as described in Fig. 4. The NanoTAC (+L) group presented markedly greater percentages of surface CRT-expressing tumor cells (CD45^−^CRT^+^), accompanied by substantial extracellular release of HMGB1 and HSP70 (Fig. 5a and Supplementary Fig. 29). In addition, the intratumoral quantities of mature DCs and CTLs increased by 1.42- and 1.57-fold and 1.55- and 1.73-fold, respectively, in the mice treated with NanoTAC (+L) compared with those treated with VPF (+L) and NanoTAC (−L), whereas the population of regulatory T (T_reg_; CD3^+^CD4^+^CD25^+^) cells decreased to 44.81% and 72.01%, respectively (Fig. 5b and Supplementary Fig. 30). Finally, tumors from NanoTAC (+L)-treated mice presented elevated levels of proinflammatory cytokines, including IL-18, IL-1β, TNF-α and IFN-γ, along with reduced levels of the anti-inflammatory cytokine IL-10 (Fig. 5c).^71^ These findings demonstrate that NanoTAC-mediated photoimmunotherapy reshapes the TME by recruiting effector cells and excluding immunosuppressive cells, which in turn facilitates antitumor immunity.

Afterward, adaptive immunity conferring immunological memory was verified in a tumor rechallenge model. Mice that achieved CR of primary TNBC by NanoTAC (+L) treatment were further inoculated with 4T1 cells into the mammary fat pad on the contralateral side. As expected, rechallenged TNBC growth was significantly inhibited in CR mice compared with that in naive mice (Fig. 5d). Rechallenged CR mice also presented marked downregulation of CD62L in splenic T cells, indicating an increase in the number of effector/memory T cells (T_em_), a cardinal mediator of adaptive immunity responsible for long-term resistance to the recurrence of previously encountered tumors (Fig. 5e).^72^ Moreover, the serum levels of TNF-α, IFN-γ, and IL-6 were 1.4-, 1.33-, and 1.32-fold greater, respectively, in NanoTAC (+L)-treated CR mice than in naive mice on day 15 after tumor rechallenge (Fig. 5f). These results suggest that NanoTAC (+L) activates both innate and adaptive immunity through enhanced PDT-induced pyroptosis via PROTAC-driven metabolic reprogramming, thereby eradicating primary TNBC and preventing recurrence.

### Prevention of metastatic TNBC formation

**Fig. 6:**
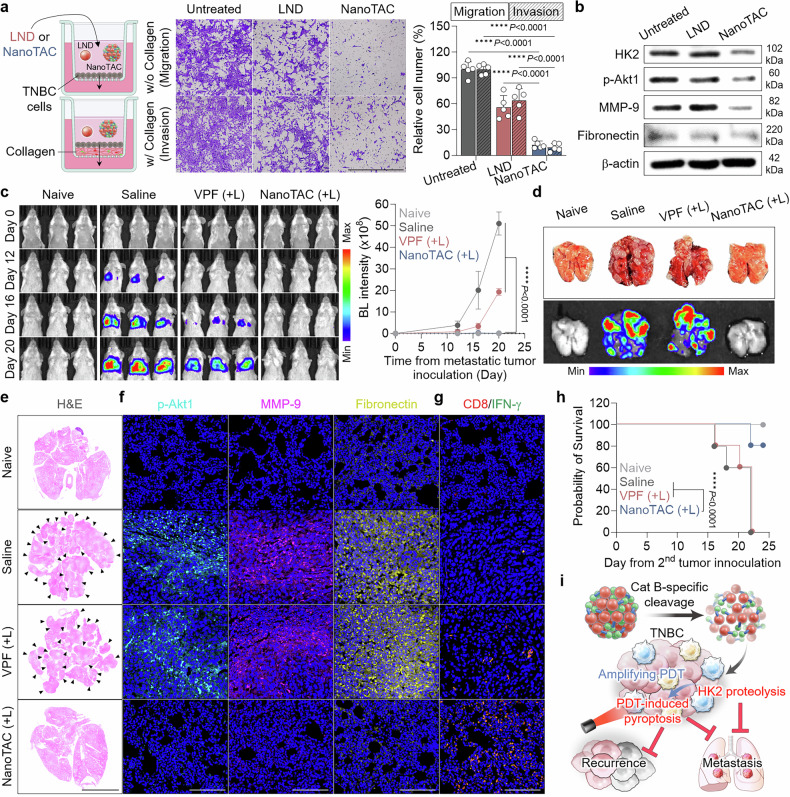
Prevention of metastatic TNBC formation by NanoTAC. **a** Schematic illustration of the migration and invasion assays, along with representative images (left) and quantitative data (right) of 4T1 cells incubated with lonidamine (LND) or NanoTAC in the absence or presence of collagen. The quantitative data are presented as the means ± SD (n = 5). Scale bar, 500 μm. **b** Expression levels of HK2 and cell motility-related proteins in 4T1 cells treated with LND or NanoTAC. **c** Time-course BL images of naive mice and pulmonary metastasis model mice treated with saline, VPF (+L), or NanoTAC (+L). The quantitative luminescence intensity in the tumor region is presented as the mean ± SD (n = 5). **d** Representative optical and BL images of harvested lungs on day 15 after metastatic tumor inoculation. Pulmonary tissues were stained with **e** H&E or antibodies against **f** p-Akt1, MMP-9, fibronectin, or **g** CD8/IFN-γ. Scale bars, 5 mm (H&E) and 50 μm (immunostaining). **h** Survival curves of mice following metastatic tumor inoculation (n = 5). **i** Illustration of NanoTAC-mediated photoimmunotherapy. Statistical significance was determined by one-way ANOVA with the Tukey‒Kramer post hoc test (**a**, **c**) and the log-rank test (**h**). The schematic illustration in (**a**) was created via BioRender.com

Finally, we explored whether NanoTAC-mediated photoimmunotherapy could also suppress TNBC metastasis. Given the crucial role of HK2 in tumor cell migration and invasion, Transwell assays were first performed.^73^ Downregulation of cellular HK2 by NanoTAC (without photoirradiation) reduced migratory and invasive phenotypes, as indicated by fewer 4T1 cells crossing from the upper to the lower chamber in the absence (migration) or presence (invasion) of collagen (Fig. 6a).^74,75^ Notably, compared with the conformational HK2 blockade by lonidamine, the TPD of NanoTAC significantly inhibited tumor cell migration and invasion.^76^ These findings are supported by western blot analyses, which revealed a pronounced reduction in phosphorylated Akt1 (p-Akt1), MMP-9 and fibronectin due to effective HK2 degradation in tumor cells treated with NanoTAC relative to those in the lonidamine-treated group (Fig. 6b). HK2 overexpression was previously reported to enhance tumor cell motility by activating the Akt1/MMP-9/fibronectin signaling pathways.^77^

The in vivo antimetastatic effect was evaluated in bilateral murine tumor models. Following systemic administration of NanoTAC or VPF into orthotopic TNBC mice and subsequent laser irradiation 3 h after treatment, 4T1 cells were injected via the intravenous route. Unlike the recurrence cancer model shown in Fig. 5, this model differs in that the TNBC cells administered to induce secondary tumors are exposed to systemically circulating NanoTAC (Supplementary Fig. 31). Real-time BL imaging demonstrated notable regression of pulmonary metastatic TNBC in the NanoTAC (+L) group compared with the other groups, which exhibited substantial tumor growth (Fig. 6c). As a consequence, NanoTAC (+L) treatment significantly reduced the number of tumor nodules in the lung tissues (Fig. 6d). Histological analyses revealed a considerable decrease in metastatic TNBC formation in the NanoTAC (+L)-treated group, whereas pulmonary metastasis was observed in widespread areas in the saline and VPF (+L) groups (Fig. 6e). Additionally, pulmonary tissues from mice treated with NanoTAC (+L) presented low expression levels of p-Akt1, MMP-9 and fibronectin, in stark contrast to those of the other treatment groups (Fig. 6f and Supplementary Fig. 32). Considerably elevated numbers of IFN-γ-expressing CD8-positive cells were further observed in lung tissues from the NanoTAC (+L) group, suggesting that CTL recruitment was driven by the in vivo vaccine effect after primary tumor eradication via pyroptosis (Fig. 6g and Supplementary Fig. 33).^78,79^ In line with these results, treatment with NanoTAC (+L) significantly improved the survival outcomes of mice with metastatic tumors (Fig. 6h). Figure 6i presents a summary of the therapeutic mechanisms of NanoTAC-mediated photoimmunotherapy. Following tumor-specific Cat-B cleavage, NanoTAC induces potent pyroptosis through PDT integrated with HK2 proteolysis, leading to complete regression of refractory TNBC and suppression of recurrence and metastasis. HK2 degradation also reduces tumor cell motility, which contributes to the antimetastatic effect.

## Discussion

To address long-standing challenges in the clinical translation of drug delivery strategies, we developed supramolecular nanoassemblies formed solely through intermolecular interactions. The drug conjugates proposed in the present study exhibit a typical prodrug structure consisting of active ingredients and a biomarker-cleavable linker, which enables selective drug release at desired targets.^80–82^ However, the incorporated PROTAC and photosensitizer have structures that appropriately balance intermolecular forces, thereby promoting spontaneous self-assembly of the system without requiring any additional carrier materials.^36,83^ This formulation holds substantial potential to overcome the bottlenecks faced by prodrug and nanomedicine approaches, of which only a small fraction of the numerous candidates have received clinical approval.

In recent decades, the rational design of prodrugs has been regarded as a promising strategy to circumvent the limitations of anticancer drugs associated with ADMET (absorption, distribution, metabolism, excretion, and toxicity) issues, such as poor solubility, low tissue permeability, chemical instability, and severe adverse effects.^84^ Nevertheless, small-molecule prodrugs suffer from rapid in vivo clearance and premature degradation, resulting in reduced therapeutic efficacy.^82^ An alternative approach, which involves covalently conjugating prodrugs to delivery materials, may enhance their pharmacokinetics (PK) but has failed to ensure antitumor effects in patients as a result of limited drug-loading capacity.^85–88^ For these reasons, a large proportion of prodrugs in the current industry aim mainly to improve the solubility or membrane permeability of existing drugs rather than to achieve purposes beyond bioavailability (e.g., reducing side effects or enabling targeted delivery) owing to difficulties in implementation.^89^

Although traditional nanomedicines (e.g., polymeric nanoparticles, liposomes, micelles, and inorganic nanoparticles) have been extensively employed to load and deliver anticancer drugs,^90^ the most critical problem is that many drugs are not amenable to carrier materials that rely on noncovalent interactions for encapsulation.^35^ This results in low drug-loading content, which contributes to several technical and practical shortcomings: (i) a high tendency to crystallize during long-term storage, (ii) premature payload leakage in systemic circulation or insufficient drug release at the target site, and (iii) overt or potential adverse reactions caused by excessive carrier material.^91^ Furthermore, their structure and preparation processes are quite complex, making it difficult to optimize protocols for quality control and large-scale industrial production.^92^

In marked contrast, the drug conjugates developed in this work are composed of approximately 60% peptide structure and synthesized through a simple two-step amide coupling reaction, allowing for scale-up and quality control in a manner similar to that of small-molecule drugs. By omitting the use of carrier materials, NanoTAC achieved a large drug-loading capacity of 78.55%, comprising 29.41% photosensitizer and 49.14% PROTAC. We have also previously established that nanoassemblies constructed via a similar approach exhibit excellent long-term storage stability, as indicated by the high reproducibility of particle characteristics after reconstitution in aqueous solution from lyophilized powder stored for a year under low, room, and accelerated temperature conditions.^93^

As a therapeutic strategy, NanoTAC aims to induce cancer metabolic reprogramming through TPD, thereby enhancing the efficiency of PDT in promoting pyroptotic cell death. During accelerated tumor growth, oxygen consumption surpasses supply, making hypoxia an intrinsic feature of many malignant solid tumors.^94^ Consequently, PDT efficiency is inevitably constrained in such a hypoxic TME, as the photosensitizer requires molecular oxygen in its ground triplet state (^3^O_2_) to generate ROS.^95^ Under hypoxic conditions, tumor cells increasingly rely on glycolysis for energy production, where the resulting increase in proliferation depletes nutrients needed by infiltrating effector T cells and impairs their functionality through the accumulation of immunosuppressive metabolites.^96^ While glycolytic inhibition typically increases cellular oxygen consumption, the HK2 inhibitor LND reduces overall oxygen consumption by suppressing both glycolysis and mitochondrial respiration.^97,98^ Therefore, LND is a promising compound for reducing oxygen consumption and reversing the immunosuppressive TME, potentially maximizing the efficacy of photoimmunotherapy. Nevertheless, given that the TME persistently maintains an immune-exclusive phenotype via homeostatic regulation and metabolic adaptation, discovering therapeutic modalities capable of inducing durable effects remains essential.^99^

To this end, we leveraged PROTAC technology because its recyclable mechanism is expected to effectively counter the PDT-resistant nature of the TME, unlike other small-molecule inhibitors.^99–101^ This study represents the first implementation of such a therapeutic strategy within the framework of supramolecular systems chemistry, in contrast to previous reports utilizing conventional nanoplatforms coloaded with photosensitizers and small-molecule glycolysis inhibitors.^29,33,102,103^ Furthermore, we demonstrated that converting LND into a PROTAC structure enables the sustained degradation of intracellular target proteins. Our investigation also extended beyond the therapeutic outcomes of PDT combined with the regulation of glycometabolism to reveal changes in various indicators (e.g., DAMPs, immune cells, and cytokines) associated with the cancer-immunity cycle.

Existing PROTAC research has focused primarily on identifying oncogenic targets for cancer therapy and optimizing chemical design to increase TPD efficiency.^100,104^ However, their toxic potential due to indiscriminate proteolysis underscores the need for methodologies that can precisely control PROTAC activity.^105^ NanoTAC addresses this issue by silencing PROTAC function by blocking the proteasome-recruiting moiety via a biomarker-cleavable linker, thereby enabling the selective release of the active PROTAC at target sites. Given the versatility of this design, which is capable of incorporating other moieties that target oncogenic, metabolic, or immunosuppressive proteins, our study provides a generalizable platform for broadening the application of PROTACs in cancer therapy.

A limitation of this study is the lack of direct comparisons with clinically relevant indications. To date, no registered clinical trials have specifically evaluated the use of PDT for TNBC. Nonetheless, ten clinical cases of PDT in breast cancer have been reported, involving photodynamic devices and various photosensitizers, including verteporfin. As these cases have emerged relatively recently, interest in applying PDT to breast cancer is increasing, likely because patients with superficial tumors are amenable to local therapy.^106^ This trend underscores the potential for future clinical application of PDT in TNBC. In this context, NanoTAC may represent a viable strategy for translating PDT into clinical practice for TNBC.

## Materials and methods

### Preparation of drug conjugates

Initially, 300 mg of N-terminal acetylated and C-terminal aminated KRRPIYPALAK(Fmoc) (0.19 mmol; Peptron) and 137 mg of VPF (0.19 mmol; Frontier Scientific) were reacted in anhydrous DMF (Sigma Aldrich) containing 147 mg of 1-ethyl-3-(3-dimethylaminopropyl)carbodiimide (EDC; 0.95 mmol; Sigma Aldrich), 109 mg of N-hydroxysuccinimide (NHS; 0.95 mmol; Sigma Aldrich) and 65 mg of N,N-diisopropylethylamine (DIPEA; 0.5 mmol; Sigma Aldrich) at room temperature for 3 h. To remove the remaining catalysts, the solution was passed through a Sep-Pak^®^ C18 cartridge (Cat. No. 186006325, Waters) and then incubated in piperidine/DMF (1:4, v/v) for 20 min to deprotect the Fmoc group. The resulting 268 mg of VPF-KRRPIYPALAK (0.13 mmol) was reacted with 41.75 mg of lonidamine (0.13 mmol; Cat. No. HY-B0486, MedChemExpress) in anhydrous DMF in the presence of 125 mg of EDC (0.65 mmol) and 75 mg of NHS (0.65 mmol) for 4 h. The final product, VPF-KRRPIYPALAK-lonidamine, was then purified via liquid chromatography‒mass spectrometry (LC/MS; Agilent Technologies). All intermediates and the final product were characterized by determining their mass‒charge ratio (m/z) and purity via LC‒MS and by analyzing hydrogen-1 nuclei (^1^H) via NMR (DD2 400 MHz FT NMR, Agilent Technologies).

### Physicochemical characterization of NanoTAC

The size distribution and polydispersity index of NanoTAC in mouse serum, along with its zeta potential in distilled water, were assessed via a Zetasizer (Malvern Panalytical). The morphology in distilled water was observed via transmission electron microscopy (TEM; Philips). UV‒vis and fluorescence spectra were measured via a UV‒vis spectrometer (Agilent Technologies) and a fluorescence spectrometer (Hitachi), respectively. ROS generation was quantified via a bleaching assay using 1,3-diphenylisobenzofuran (DPBF; Cat. No. 105481; Sigma Aldrich). Equimolar amounts of NanoTAC or VPF dispersed in DPBF solution were irradiated with a 671 nm laser (Shanghai Dream Laser Technology) at a power of 40 mW, followed by analysis via a UV‒vis spectrometer. The ROS quantum yield (Φ) of NanoTAC was calculated relative to VPF (Φ = 0.78) according to the following equation.^107^

$${\Phi }_{\text{NanoTAC}}={\Phi }_{\text{VPF}}\times \left(\frac{{\text{A}}_{\text{VPF}}}{{\text{A}}_{\text{NanoTAC}}}\right)\times \left(\frac{{\text{S}}_{\text{NanoTAC}}}{{\text{S}}_{\text{VPF}}}\right)$$

A_VPF_ and A_NanoTAC_ represent the absorbance at the excitation wavelength (671 nm), whereas S_VPF_ and S_NanoTAC_ denote the slopes of the absorbance intensity over time. To assess target biomarker-specific cleavage, NanoTAC at pH 5.5 2-(N-morpholino)ethanesulfonic acid (MES) buffer was incubated at 37 °C with Cat-B (1, 2, 5 or 10 μg; Cat. No. 953CY, R&D systems), Cat-B with Z-FA-FMK (Cat. No. HY-P0109A, MedChemExpress), Cat-D (Cat. No. 1014-AS, R&D Systems), Cat-E (Cat. No. 1294-AS, R&D Systems), Cat-L (Cat. No. 952-CY, R&D Systems), MMP-9 (Cat. No. 911-MP, R&D Systems) or CASP3 (Cat. No. 707-C3/CF, R&D Systems), followed by LC/MS analysis. To determine the catalytic efficiency (*k*_cat_/*K*_M_), NanoTAC (5, 10, 20, 40, and 80 μM) was dispersed in pH 5.5 MES buffer containing 5 μg of Cat-B. During incubation at 37 °C, a portion was collected and analyzed via HPLC. The cleavage rate of each sample was calculated by comparing the peak area to that of an equivalent amount of NanoTAC prior to enzyme incubation. The resulting data were plotted, and *k*_cat_/*K*_M_ was determined via Prism 10 software (GraphPad).

### Molecular dynamics (MD) simulation

MD simulations were performed via GROMACS software version 2021.2. The NanoTAC structure was initially designed via ChemDraw Professional 20.1.1.125 (PerkinElmer). All the molecules were parameterized according to the CHARMM36m force field, with the corresponding parameters generated via the CHARMM-GUI web server. The simulation system was solvated via the TIP3P water model, and counterions (Na⁺ and Cl^−^) were added to neutralize the overall charge of the system. The simulations were conducted over a 50 ns timescale. Short-range van der Waals and electrostatic interactions were calculated using a cutoff distance of 1.4 nm. Long-range electrostatic interactions were computed via the particle‒mesh-Ewald (PME) method with a Fourier spacing of 0.24 nm and fourth-order interpolation to increase accuracy. All bonds involving hydrogen atoms were constrained via the LINear Constraint Solver (LINCS) algorithm. Temperature control was maintained via the V-rescale thermostat, whereas pressure was controlled via the Berendsen barostat during equilibration and the Parrinello–Rahman barostat during the production phase. These simulations were conducted at a constant temperature of 300 K and pressure of 1 bar to replicate physiological conditions. The resulting simulation trajectories were visualized and analyzed via PyMOL software (version 2.1.0; Schrödinger).

### In silico analysis of NanoTAC

Following the MD simulations, the electrostatic and interaction profiles of the drug conjugates were further analyzed. The trajectory data were imported into Discovery Studio 2022 software (v22.1.0; BIOVIA) for postsimulation analysis. The Chemistry at Harvard Molecule Mechanics (CHARMm) force field was applied via the “Apply Force Field” module, and interpolated charges were assigned via the Momany–Rone method. Nonbonded interactions, including hydrogen bonds, electrostatic forces, and hydrophobic contacts, were visualized and quantified via the nonbond interaction module. These interactions were evaluated between molecules, including hydrogen-involving contacts, within a 4 Å cutoff distance. Additionally, the solvent accessibility surface area (SASA) was computed via a probe radius of 1.40 Å with 240 grid points per atom. The Lennard‒Jones short-range (LJ‒SR) interaction energy between NanoTAC molecules was analyzed via the gmx energy utility in GROMACS (version 2021.2).

### Molecular binding simulation

The molecular structures of NanoTAC, LND, and PROTAC were illustrated via ChemDraw Professional 20.1.1.125 (PerkinElmer). Molecular binding was performed with AMDock 1.5.2 (Assisted Molecular Docking) software against the HK2 and VHL proteins (PDB: 2NZT, 1LM8), utilizing the CHARMm (Chemistry at Harvard Molecule Mechanics) force field. All proteins were prepared via the Prepare Protein module of Discovery Studio 2022 software. The binding site was identified on the basis of the active site of the ligand present in the existing PDB. During the docking process, ten poses were generated, and the most favorable pose was selected and visualized via PyMOL. In addition, the binding affinity was calculated to evaluate the strength of the interactions between the protein and the ligand.^108^ After the docking process, 2D diagram interaction analysis was performed via Discovery Studio software. Following the binding analysis process, MD simulations were performed via the standard dynamics cascade protocol in Discovery Studio software. The simulations were conducted under an isothermal–isobaric ensemble, incorporating the generalized distance-dependent dielectric implicit solvent model, with a production run of 100 ps. The resulting MD trajectories were analyzed via the TRAnsient Pockets in Proteins (TRAPP) web server to evaluate the root-mean-square deviation (RMSD) between the ligand and the HK2 and VHL proteins. All analyses were carried out via the default parameters provided by the TRAPP platform.^109^ After the ligand-bound structures of HK2 and VHL were uploaded to TRAPP, the generated trajectory files were submitted for interaction analysis. During this process, the distance considered around the ligand center was set to 8 Å.

### Binding kinetics analysis

The binding kinetics were analyzed via a GatorPrime instrument (Gator Bio). PROTAC, which was cleaved from NanoTAC following reaction with Cat-B, was purified via HPLC. His probes (Gator Bio) were activated in 200 μL of PBS (pH 7.4) for 10 min, followed by baseline stabilization in 200 µL of PBS at 35 °C and 1000 rpm for 30 s. HK2 (0.16 μM, Cat. No. CS-P703943, ChemScene) or VHL (0.2 μM, Cat. No. CS-P76124, ChemScene) was reacted with the His probes in PBS for 300 s to reach binding equilibrium. The association and dissociation of PROTACs were subsequently measured in PBS over 150 s at concentrations ranging from 2.5 to 20 µM. The dissociation constant (K_D_) was calculated via GatorLaunch software (version 2.10.4.0713; Gator Bio).

### Cellular uptake

The murine mammary carcinoma cell line 4T1 was purchased from ATCC (American Type Culture Collection) and cultured in RPMI 1640 supplemented with 10% fetal bovine serum (WelGene) and 1% penicillin/streptomycin (WelGene) at 37 °C in a 5% CO_2_ atmosphere. A total of 2 × 10^5^ 4T1 cells were seeded in confocal dishes (SPL Life Science) and treated with 1 μM NanoTAC or VPF. The cells were then fixed with 4% paraformaldehyde (Biosesang) for 10 min and stained with 4′,6-diamidino-2-phenylindole (DAPI; Invitrogen) for 5 min. NIRF images were captured via confocal laser scanning microscopy (CLSM; Leica Microsystems), and the mean fluorescence intensity was quantified via ImageJ software (National Institutes of Health).

### TPD analysis

To evaluate HK2 degradation, 2 × 10^5^ 4T1 cells seeded in confocal dishes were incubated with 1 μM NanoTAC for 12 h, followed by staining with an anti-HK2 antibody (Cat. No. 2867T, Cell Signaling Technology), a fluorescent dye-conjugated secondary antibody (Cat. No. A11008, Invitrogen), and DAPI. For the mechanism studies, the cells were pretreated with CA-074-ME (Cat. No. HY-103350, MedChemExpress), pevonedistat (Cat. No. HY-70062, MedChemExpress), or epoxomicin (Cat. No. HY-13821, MedChemExpress) for 6 h prior to NanoTAC treatment. Intracellular HK2 expression was visualized via CLSM.

The effects of intracellular HK2 degradation were investigated by quantifying lactate and oxygen levels in 4T1 cells treated as described above. Extracellular lactate in the culture medium was measured via a Lactate-Glo™ Assay Kit (Cat. No. J5021, Promega), whereas oxygen levels were assessed via CLSM imaging after staining with the oxygen indicator tris(4,7-diphenyl-1,10-phenanthroline)ruthenium(II) dichloride (RDPP; Cat. No. HY-W074143, MedChemExpress). To monitor changes in oxygen availability under hypoxia, 4T1 cells treated with VPF or NanoTAC were incubated for 12 h in a Galaxy 48R incubator (Eppendorf) maintained at an atmosphere of 1% O_2_, 5% CO_2_, and 94% N_2_, followed by staining with RDPP.

### ROS generation and cytotoxicity

Diacetyldichlorofluorescein (DCFH-DA; Cat. No. 4091-99-0, Sigma Aldrich) was used to detect intracellular ROS. Under normoxic or hypoxic conditions, a total of 5 × 10^5^ 4T1 cells seeded in confocal dishes were treated with 1 μM VPF or NanoTAC for 12 h. The cells were then incubated with DCFH-DA for 30 min and subsequently exposed to photoirradiation for 500 s at a power of 40 mW. NIRF images were captured via CLSM.

For cytotoxicity analysis, the cells were subjected to the same protocol and further incubated with RPMI 1640 medium supplemented with 10% Cell Counting Kit-8 (v/v; Cat. No. DJDB4000X, Vita Scientific). Cell viability was measured via a UV‒vis microplate reader (VERSAmax™) at a wavelength of 450 nm.

### Pyroptosis evaluation and coculture assay

Pyroptosis was evaluated by monitoring changes in protein expression and cellular morphology. 4T1 cells were seeded in six-well plates at a density of 5 × 10^5^ and treated with 1 μM VPF or NanoTAC for 12 h, with or without photoirradiation (40 mW for 500 s). The levels of proteins associated with pyroptosis were analyzed via western blotting with antibodies against HK2, cleaved CASP3 (Cat. No. 9661S; Cell Signaling Technology), and GSDME (Cat. No. ab215191; Abcam). Morphological changes following each treatment were observed via CLSM.

For the analysis of DAMPs, 4T1 cells seeded in six-well plates were treated as described above. Afterward, the cells were stained with a fluorescent dye-conjugated CRT antibody (Cat. No. ab196159, Abcam), followed by flow cytometry (BD Bioscience). In the culture medium, HMGB1 (Cat. No. ab18256, Abcam) and HSP70 (Cat. No. ab2787, Abcam) levels were assessed via western blotting, whereas ATP (Cat. No. S0026B, Beyotime Biotechnology), IL-18 (Cat. No. DY7625-05, R&D Systems) and IL-1β (Cat. No. MLB00C, R&D Systems) levels were quantified via commercial ELISA kits.

Animal studies were conducted in accordance with the relevant laws and institutional guidelines of the Institutional Animal Care and Use Committee at the Korea Institute of Science and Technology (KIST; IACUC approval number: 2023-013). Female BALB/c nude and BALB/c mice (7 weeks old) were acquired from Nara Biotech and maintained under pathogen-free conditions at KIST. For coculture studies, 4T1 cells treated as described above were cocultured with splenocytes isolated from BALB/c mice for 24 h. Multiparameter staining was performed with appropriate antibodies to identify the following immune cell populations: mature DCs (CD11c^+^, Cat. No. 117310, Biolegend; CD40^+^, Cat. No. 104722, Biolegend; CD86^+^, Cat. No. 105007, Biolegend), CTLs (CD45^+^, Cat. No. 109828, Biolegend; CD3^+^, Cat. No. 100217, Biolegend; CD8^+^, Cat. No. 100712, Biolegend) and regulatory T cells (CD3^+^; CD4^+^, Cat. No. 116013, Biolegend; CD25^+^, Cat. No. 101904, Biolegend). In the coculture medium, TNF-α (Cat. No. MTA00b-1, R&D Systems), IFN-γ (Cat. No. MIF00-1, R&D Systems), and IL-10 (Cat. No. M1000B-1, R&D Systems) were quantified via ELISA kits.

### Biodistribution in an orthotopic TNBC mouse model

Luciferase-expressing 4T1 cells suspended in RPMI 1640 medium were inoculated into the fourth mammary fat pad of BALB/c nude mice (2 × 10^5^ cells per mouse). The tumor volume was calculated via the following formula: longest diameter × (shortest diameter)^2^ × 0.53 mm^3^. When the tumors reached approximately 50 mm^3^, an equivalent dose of free VPF or NanoTAC, which was based on 5 mg/kg VPF, was administered intravenously. In vivo BL and NIRF images were captured via an IVIS system (PerkinElmer). For BL imaging, 3 mg of D-luciferin (Cat. No. eLUCK-100, Gold Biotechnology) was injected intraperitoneally into the mice. Luminescence and fluorescence intensities within the tumor regions were quantified via LivingImage^®^ software (PerkinElmer). Tumor tissues resected 24 h after treatment were stained with antibodies against HK2 or HIF-1α (Cat. No. PA1-16601, Invitrogen), and the intratumoral expression of these proteins was observed via CLSM. For PK analysis, blood samples were collected at the indicated time points, centrifuged at 2200 rpm for 15 min, and analyzed for fluorescence intensity using a microplate reader (GloMax®, Promega).

To assess the cleavage behavior of NanoTAC under various in vivo conditions, lysates of major organs (liver, lung, kidney, spleen, and heart) as well as tumor tissues were collected from TNBC mice. These samples were incubated with NanoTAC for 24 h, followed by HPLC analysis. Cathepsin B levels were analyzed by western blot using an appropriate antibody (Cat. No. sc-365558; Santa Cruz Biotechnology).

### Tumor inhibition and antitumor immunity in a TNBC mouse model

Luciferase-expressing 4T1 tumor-bearing BALB/c mice were divided into five groups: (i) saline, (ii) laser only (laser), (iii) VPF (+L), (iv) NanoTAC (−L), and (v) NanoTAC (+L). When the tumor volume was approximately 50 mm^3^, an equivalent 5 mg/kg VPF dose of NanoTAC or free VPF was intravenously administered, followed by photoirradiation of the tumor area at 200 mW for 15 min. BL imaging was conducted every 4 days following the intraperitoneal injection of D-luciferin. The tumor volumes and body weights were measured every 2 days. Mice with tumor volumes exceeding 500 mm^3^ were considered deceased.

Lactate levels in tumor supernatants were quantified via the Lactate-Glo™ Assay Kit. Intratumoral ROS generation following laser irradiation (200 mW, 15 min) was visualized by staining with dihydroethidium (DHE; Cat. No. HY-D0079, MedChemExpress). To assess the influence of lactate in the TME on immune cell function, spleens were harvested from 7-week-old female BALB/c mice, and single-cell suspensions were prepared by mechanical dissociation through a cell strainer (Cat. No. 431750, Corning). Following red blood cell lysis (Cat. No. 420301, BioLegend), T cells were isolated via negative selection with the CD8^+^ T-Cell Isolation Kit (Cat. No. 130-104-075, Miltenyi Biotechnology). The isolated cells were cultured in RPMI 1640 medium supplemented with recombinant mouse IL-2 (3 U/mL; Cat. No. 212-12-20UG; Gibco) and 2-mercaptoethanol (0.05 mM; Cat. No. 21985023; Thermo Fisher Scientific). After 12 h of activation, the T cells were incubated with tumor supernatants for an additional 24 h, stained with fluorescent dye-conjugated antibodies against PD-1 (Cat. No. 135213, BioLegend) and TIM3 (Cat. No. 134003, BioLegend), and analyzed by flow cytometry.

Following treatment of the TNBC mouse model described above, tumor tissues were collected on day 3 for immune cell population analyses. Single cells were isolated from the tumor samples via a tumor dissociation kit (Cat. No. 130-096-730; Miltenyi Biotechnology). An equal number of cells were incubated with FcBlock (Cat. No. 553142, BD Biosciences) for 5 min to prevent nonspecific IgG binding and then stained with appropriate antibodies to identify the populations of CRT-positive tumor cells (CD45^−^CRT^+^), mature DCs, CTLs, and regulatory T cells. As in the coculture study, HMGB1 and HSP70 in the tumor supernatants were assessed by western blot, while IL-18, IL-1β, TNF-α, IFN-γ, and IL-10 were measured via ELISA kits.

Sixteen days post-treatment, TNBC mice were inoculated with 4T1 cells (2 × 10^5^ cells per mouse) into the fourth mammary fat pad opposite the primary TNBC site. Rechallenged tumor volumes were measured every 2 days. Fifteen days after tumor rechallenge, the population of splenic T_em_ cells (CD3^+^; CD8^+^; CD44^+^ [Cat. No. 103016, Biolegend]; CD62L^low^ [Cat. No. 104407, Biolegend]) was analyzed via flow cytometry. Finally, the serum levels of IFN-γ, IL-6, and TNF-α were quantified via ELISA.

### Evaluation of antimetastatic effects in a bilateral tumor model

4T1 tumor-bearing BALB/c mice in the (i) naive, (ii) saline, (iii) VPF (+L), and (iv) NanoTAC (+L) groups were treated according to the protocol described above. A total of 1 × 10^6^ luciferase-expressing 4T1 cells were subsequently intravenously administered to all the groups except the naive group. BL imaging was performed every 4 days following the intraperitoneal injection of D-luciferin. After 2 weeks, the metastatic lesions in the lung tissues were visualized via hematoxylin and eosin (H&E) staining. Protein markers related to the antimetastatic effect of HK2, including phosphorylated Akt1 (Cat. No. D7F10, Cell Signaling), MMP-9 (Cat. No. MA5-15886, Invitrogen), fibronectin (Cat. No. PA5-29578, Invitrogen), CD8 (Cat. No. 100712, Biolegend) and IFN-γ (Cat. No. 505808, Biolegend), were assessed via immunofluorescence staining of pulmonary tissues with appropriate antibodies.

### Statistics and reproducibility

All the experiments were conducted with a minimum of three replicates, yielding consistent results. The number of experimental replicates (n) is indicated in the figure legends. All the results are presented as the means ± standard deviations (SDs). Statistical analyses were performed via Prism 10 software (GraphPad). The statistical significance of differences between two groups was analyzed via Student’s t-test. For comparisons among more than two groups, one-way analysis of variance (ANOVA) was used, followed by Tukey‒Kramer post hoc testing for multiple comparisons. Survival data were plotted via Kaplan‒Meier curves and analyzed with the log-rank test. The detailed statistical methods and *P* values are specified in the figure legends. *P* values of <0.05^*^, <0.01^**^, <0.001^***^, and <0.0001^****^ were considered statistically significant, and “ns” denotes not significant (P > 0.05). Data collection and analysis were conducted in a blinded manner. Investigators were blinded to group allocation during both the experiments and outcome assessments.

## Supplementary information

Supplementary Materials

Uncropped Western Blot Images

## Data Availability

All relevant data are available within the paper and its Supplementary Information files or are available from the corresponding authors upon reasonable request. Source data are provided with this paper. Uncropped original full Western blot images are provided in a separate file.
