# Supplementary material for: Reprogramming of cancer metabolism via photoresponsive nano-PROTAC enhances pyroptosis-mediated immunotherapy
Source: Signal Transduct Target Ther. 2025 Sep 26;10:310. doi: 10.1038/s41392-025-02405-6 (PMC12464334; doi:10.1038/s41392-025-02405-6)
Supplement: Supplementary file 1 — Supplementary Materials [file 41392_2025_2405_MOESM1_ESM.docx]

Supplementary Materials for

**Reprogramming of Cancer Metabolism via Photoresponsive Nano-PROTAC Enhances Pyroptosis-Mediated Immunotherapy**

Byeongmin Park^#^, Jiwoong Choi^#^, Jae-Hyeon Lee^#^, Yelee Kim, Woohyeong Lee, Ansoo Lee,
In-Cheol Sun, Hong Yeol Yoon, Yongju Kim, Sun Hwa Kim, Yoosoo Yang, Kwangmeyung Kim, Jooho Park^*^, Man Kyu Shim^*^

^*^Correspondence to:

Man Kyu Shim (E-mail: mks@kist.re.kr) and Jooho Park (E-mail: pkjhdn@kku.ac.kr).

**This file includes:**

Supplementary Figures 1-33


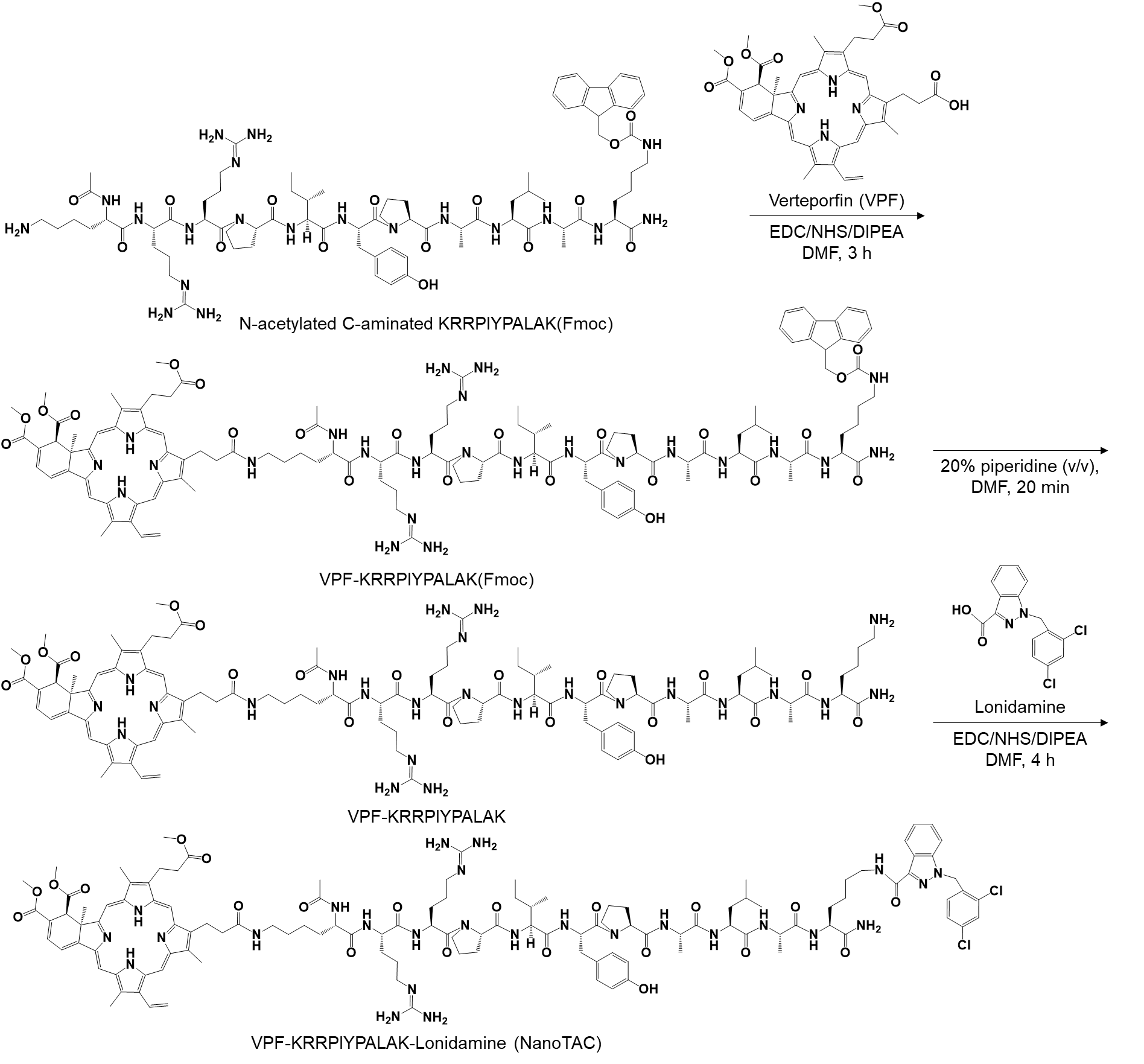


**Supplementary Figure 1. Synthetic scheme of the drug conjugate composing NanoTAC.** The photosensitizer verteporfin (VPF) was first introduced into the N-terminal acetylated and C-terminal aminated KRRPIYPALAK(Fmoc) peptide (DMF, DIPEA, 3 h) to obtain VPF-KRRPIYPALAK(Fmoc), which was then deprotected in a DMF solution containing 20% piperidine (v/v) for 20 min to yield VPF-KRRPIYPALAK. Subsequently, lonidamine was conjugated to VPF-KRRPIYPALAK (DMF, DIPEA, 4 h), resulting in VPF-KRRPIYPALAK-lonidamine (NanoTAC).


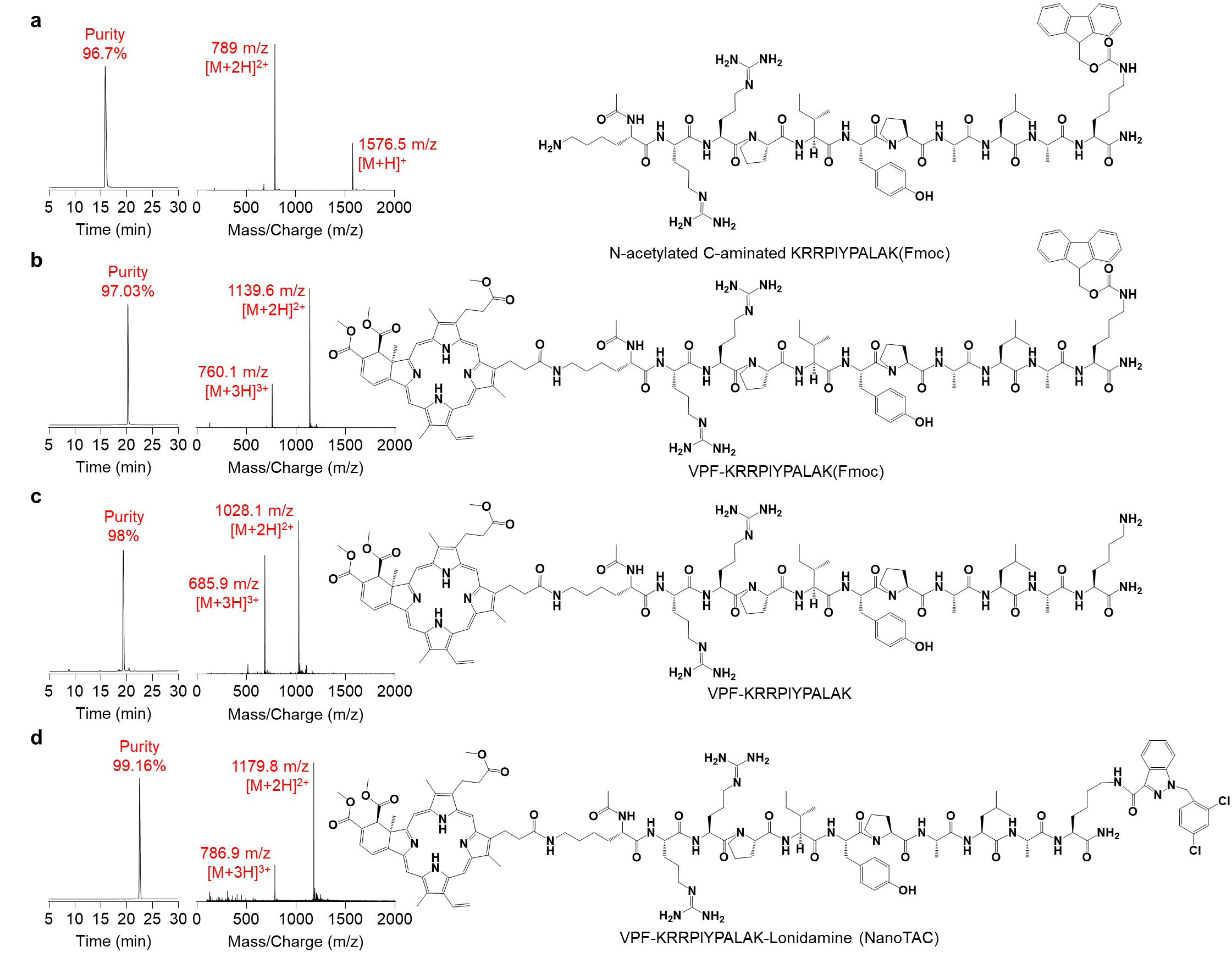


**Supplementary Figure 2.** HPLC spectrum (purity) and mass spectra of **(a)** N-terminal acetylated and C-terminal aminated KRRPIYPALAK(Fmoc) peptide (calculated for C_78_H_118_N_20_O_15_, found: 1576.5 m/z [M+H]^+^ and 789 m/z [M+2H]^2+^), **(b)** VPF-KRRPIYPALAK(Fmoc) (calculated for C_119_H_158_N_24_O_22_, found: 1139.6 m/z [M+2H]^2+^ and 760.1 m/z [M+3H]^3+^), **(c)** VPF-KRRPIYPALAK (calculated for C_104_H_148_N_24_O_20_, found: 1028.1 m/z [M+2H]^2+^ and 685.9 m/z [M+3H]^3+^) and **(d)** VPF-KRRPIYPALAK-lonidamine (calculated for C_119_H_156_N_26_O_21_, found: 1179.8 m/z [M+2H]^2+^ and 786.9 m/z [M+3H]^3+^).


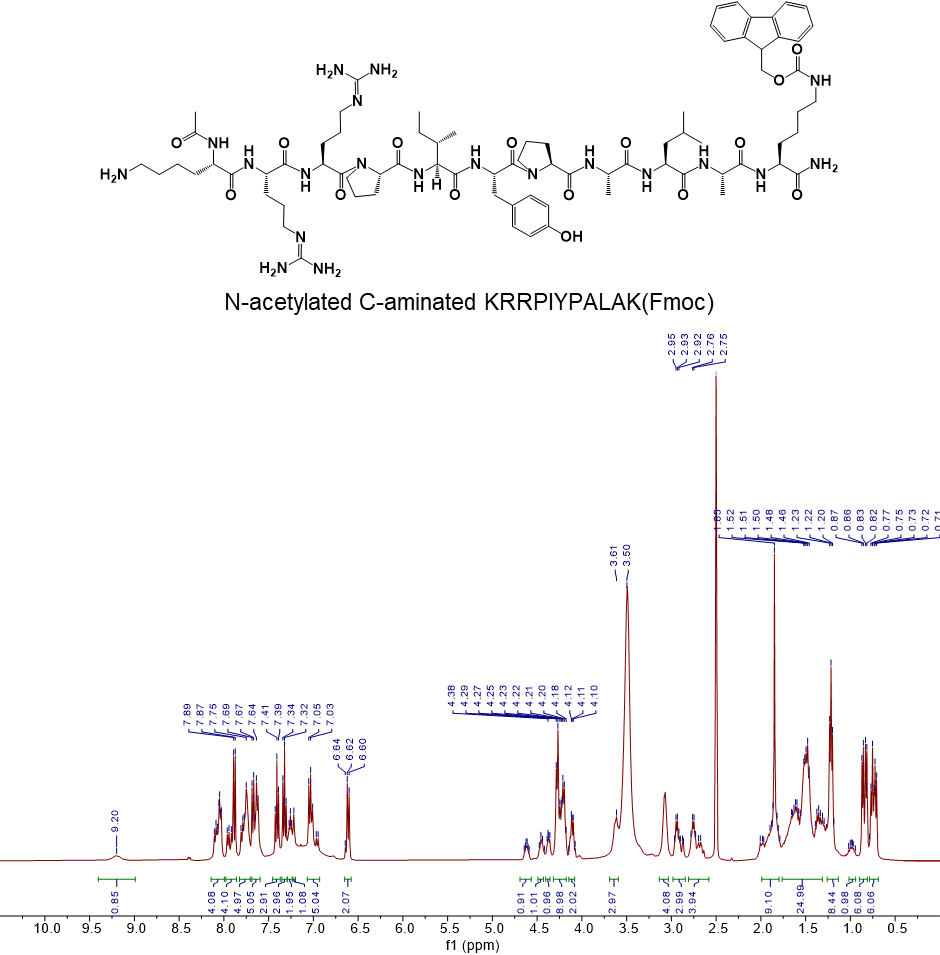


**Supplementary Figure 3.** ^1^H-NMR spectrum of N-acetylated C-aminated KRRPIYPALAK(Fmoc). ^1^H NMR (400 MHz, DMSO-d_6_) δ 9.21 (s, 1H), 8.15 – 8.00 (m, 4H), 7.99 – 7.87 (m, 4H), 7.83 – 7.71 (m, 5H), 7.70 – 7.60 (m, 5H), 7.42 (t, *J* = 7.5 Hz, 3H), 7.33 (t, *J* = 7.4 Hz, 3H), 7.27 (t, *J* = 5.5 Hz, 2H), 7.24 – 7.21 (m, 1H), 7.08 – 6.94 (m, 5H), 6.66 – 6.59 (m, 2H), 4.70 – 4.57 (m, 1H), 4.50 – 4.44 (m, 1H), 4.41 – 4.36 (m, 1H), 4.33 – 4.18 (m, 9H), 4.15 – 4.09 (m, 2H), 3.70 – 3.60 (m, 3H), 3.14 – 3.04 (m, 4H), 2.99 – 2.85 (m, 3H), 2.82 – 2.59 (m, 4H), 2.00 – 1.81 (m, 9H), 1.77 – 1.32 (m, 25H), 1.27 – 1.14 (m, 8H), 1.02 – 0.95 (m, 1H), 0.85 (dd, *J* = 15.7, 6.5 Hz, 6H), 0.80 – 0.70 (m, 6H).


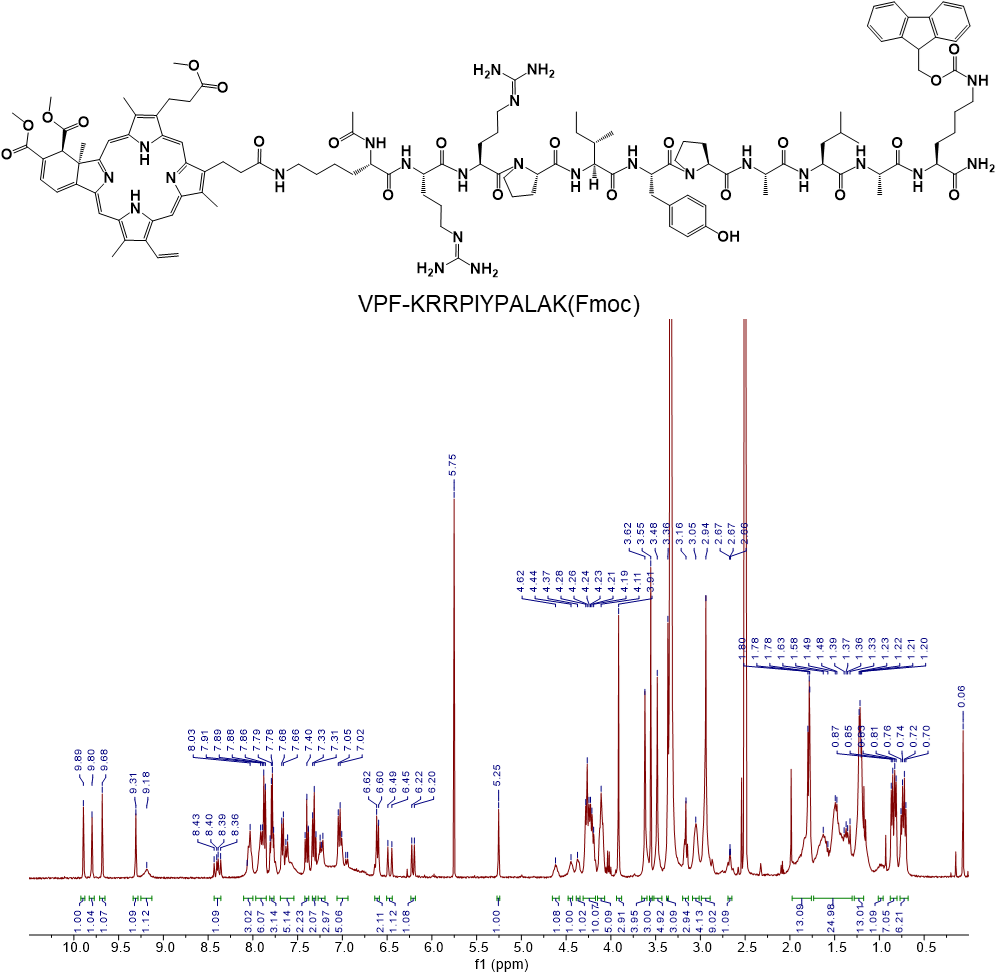


**Supplementary Figure 4.** ^1^H-NMR spectrum of VPF-KRRPIYPALAK(Fmoc). ^1^H NMR (400 MHz, DMSO-d_6_) δ 9.89 (s, 1H), 9.80 (s, 1H), 9.68 (s, 1H), 9.31 (s, 1H), 9.18 (s, 1H), 8.38 (dd, *J* = 17.6, 11.6 Hz, 1H), 8.10 – 8.00 (m, 3H), 7.97 – 7.85 (m, 6H), 7.79 (q, *J* = 5.7 Hz, 3H), 7.69 – 7.54 (m, 5H), 7.40 (t, *J* = 7.0 Hz, 2H), 7.32 (t, *J* = 7.4 Hz, 2H), 7.27 – 7.19 (m, 3H), 7.06 – 6.94 (m, 5H), 6.64 – 6.59 (m, 2H), 6.47 (d, *J* = 17.8 Hz, 1H), 6.21 (d, *J* = 11.6 Hz, 1H), 5.25 (s, 1H), 4.65 – 4.58 (m, 1H), 4.48 – 4.43 (m, 1H), 4.39 – 4.35 (m, 1H), 4.31 – 4.17 (m, 10H), 4.15 – 4.07 (m, 5H), 3.91 (s, 3H), 3.62 (s, 4H), 3.55 (s, 3H), 3.48 (s, 5H), 3.36 (s, 3H), 3.20 – 3.13 (m, 3H), 3.09 – 3.01 (m, 4H), 2.99 – 2.89 (m, 9H), 2.69 – 2.65 (m, 1H), 1.97 – 1.76 (m, 13H), 1.73 – 1.31 (m, 25H), 1.28 – 1.18 (m, 13H), 1.01 – 0.96 (m, 1H), 0.84 (dd, *J* = 15.4, 6.6 Hz, 7H), 0.77 – 0.68 (m, 6H).


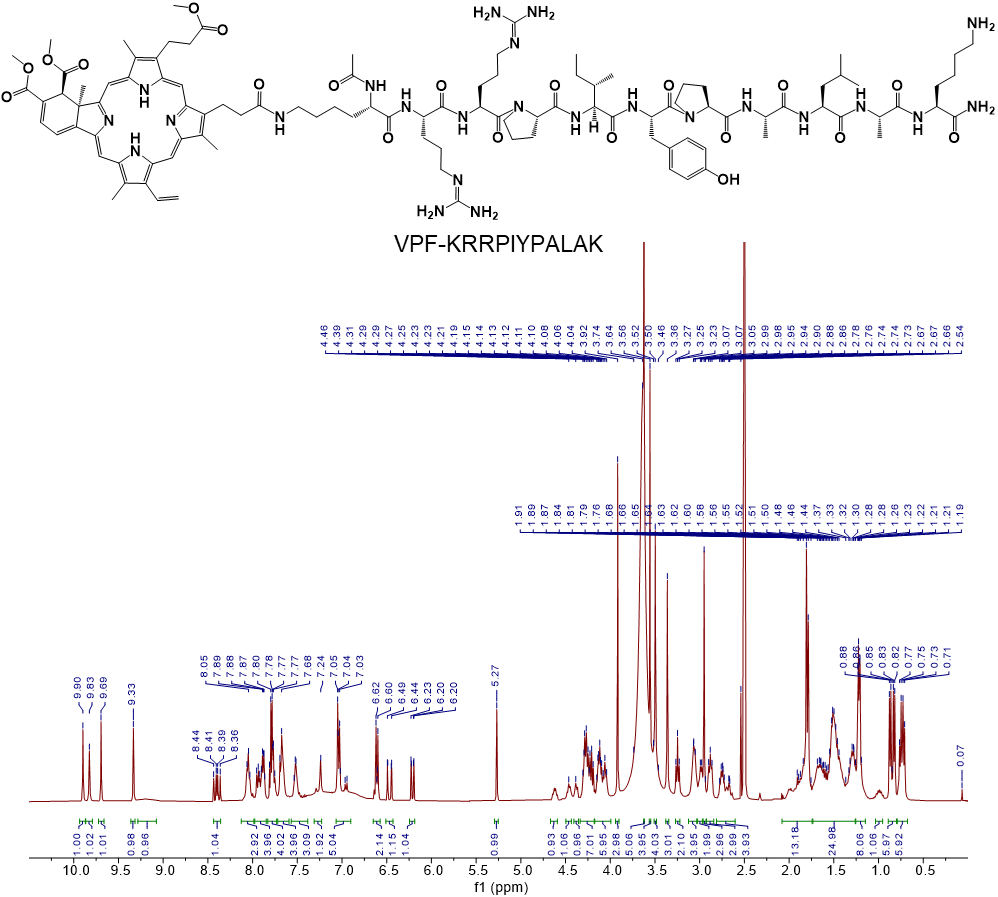


**Supplementary Figure 5.** ^1^H-NMR spectrum of VPF-KRRPIYPALAK. ^1^H NMR (400 MHz, DMSO-d_6_) δ 9.90 (s, 1H), 9.83 (s, 1H), 9.69 (s, 1H), 9.33 (s, 1H), 9.18 (s, 1H), 8.40 (dd, *J* = 17.8, 11.6 Hz, 1H), 8.13 – 7.99 (m, 3H), 7.98 – 7.85 (m, 4H), 7.83 – 7.73 (m, 4H), 7.72 – 7.59 (m, 4H), 7.57 – 7.38 (m, 3H), 7.31 – 7.23 (m, 2H), 7.07 – 6.90 (m, 5H), 6.65 – 6.57 (m, 2H), 6.47 (dd, *J* = 17.8, 1.7 Hz, 1H), 6.21 (dd, *J* = 11.6, 1.5 Hz, 1H), 5.27 (s, 1H), 4.67 – 4.59 (m, 1H), 4.50 – 4.43 (m, 1H), 4.41 – 4.36 (m, 1H), 4.34 – 4.18 (m, 7H), 4.17 – 3.99 (m, 6H), 3.92 (s, 3H), 3.62 (s, 5H), 3.56 (s, 4H), 3.50 (s, 4H), 3.36 (s, 3H), 3.25 (t, *J* = 7.5 Hz, 2H), 3.12 – 3.03 (m, 4H), 3.03 – 2.97 (m, 2H), 2.95 (s, 3H), 2.93 – 2.84 (m, 3H), 2.81 – 2.60 (m, 4H), 2.08 – 1.74 (m, 13H), 1.73 – 1.26 (m, 25H), 1.25 – 1.15 (m, 8H), 1.00 (q, *J* = 7.2 Hz, 1H), 0.85 (dd, *J* = 17.4, 6.4 Hz, 6H), 0.79 – 0.68 (m, 6H).

**
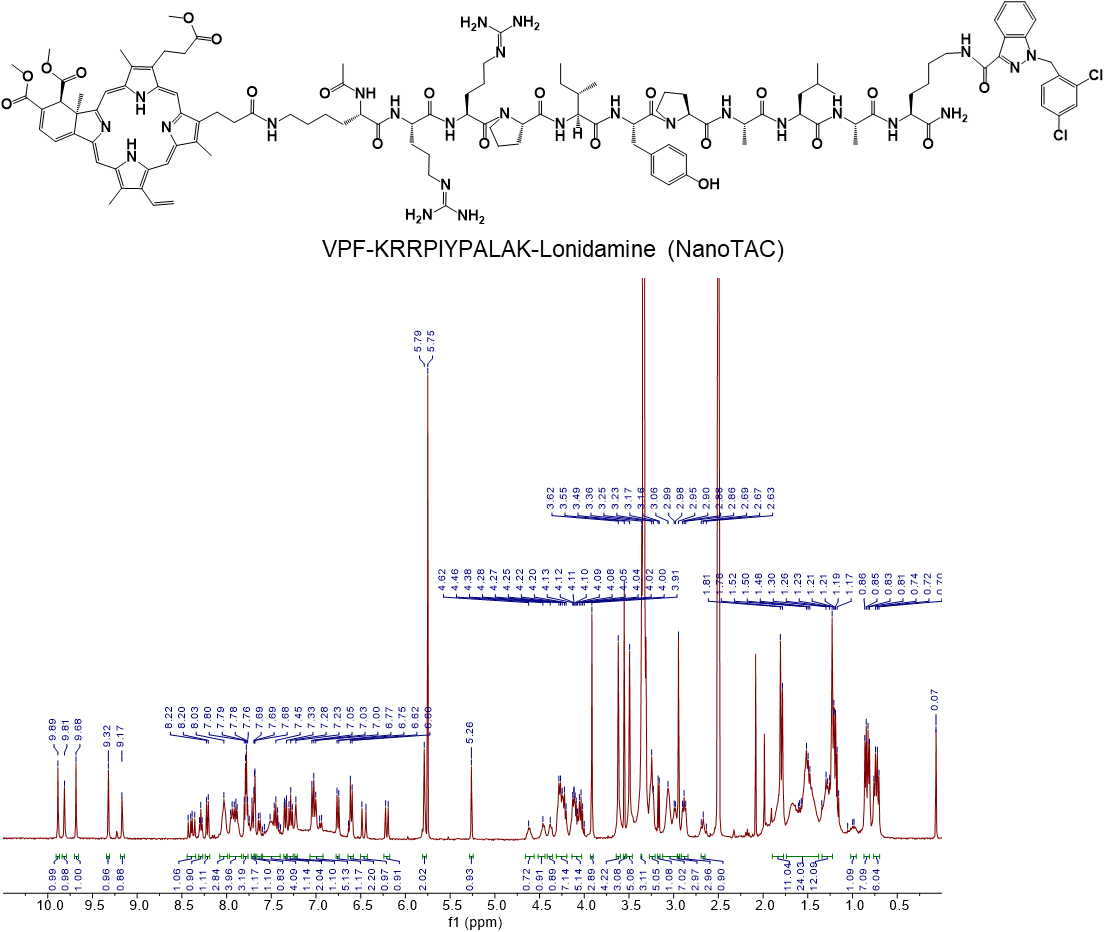
**

**Supplementary Figure 6.** ^1^H-NMR spectrum of VPF-KRRPIYPALAK-Lonidamine (NanoTAC). ^1^H NMR (400 MHz, DMSO-d_6_) δ 9.89 (s, 1H), 9.81 (s, 1H), 9.68 (s, 1H), 9.32 (s, 1H), 9.17 (s, 1H), 8.39 (dd, *J* = 17.8, 11.7 Hz, 1H), 8.29 (t, *J* = 5.9 Hz, 1H), 8.21 (d, *J* = 7.9 Hz, 1H), 8.08 – 7.99 (m, 3H), 7.97 – 7.83 (m, 4H), 7.78 (q, *J* = 5.7 Hz, 3H), 7.72 – 7.69 (m, 1H), 7.69 – 7.67 (m, 1H), 7.63 (d, *J* = 8.0 Hz, 1H), 7.60 – 7.40 (m, 4H), 7.34 (dd, *J* = 8.3, 2.1 Hz, 1H), 7.28 (t, *J* = 7.5 Hz, 2H), 7.23 (s, 1H), 7.07 – 6.92 (m, 5H), 6.76 (d, *J* = 8.3 Hz, 1H), 6.64 – 6.58 (m, 2H), 6.46 (d, *J* = 18.2 Hz, 1H), 6.21 (d, *J* = 11.9 Hz, 1H), 5.79 (s, 2H), 5.26 (s, 1H), 4.66 – 4.56 (m, 1H), 4.52 – 4.44 (m, 1H), 4.41 – 4.35 (m, 1H), 4.31 – 4.19 (m, 7H), 4.14 – 4.03 (m, 5H), 3.91 (s, 3H), 3.62 (s, 4H), 3.55 (s, 3H), 3.49 (s, 5H), 3.36 (s, 3H), 3.27 – 3.21 (m, 5H), 3.17 (d, *J* = 5.1 Hz, 1H), 3.12 – 2.96 (m, 7H), 2.95 (s, 3H), 2.92 – 2.84 (m, 3H), 2.69 – 2.65 (m, 1H), 1.90 – 1.77 (m, 11H), 1.74 – 1.38 (m, 24H), 1.34 – 1.22 (m, 12H), 1.02 – 0.96 (m, 1H), 0.85 (dd, 7H), 0.76 – 0.70 (m, 6H).

**
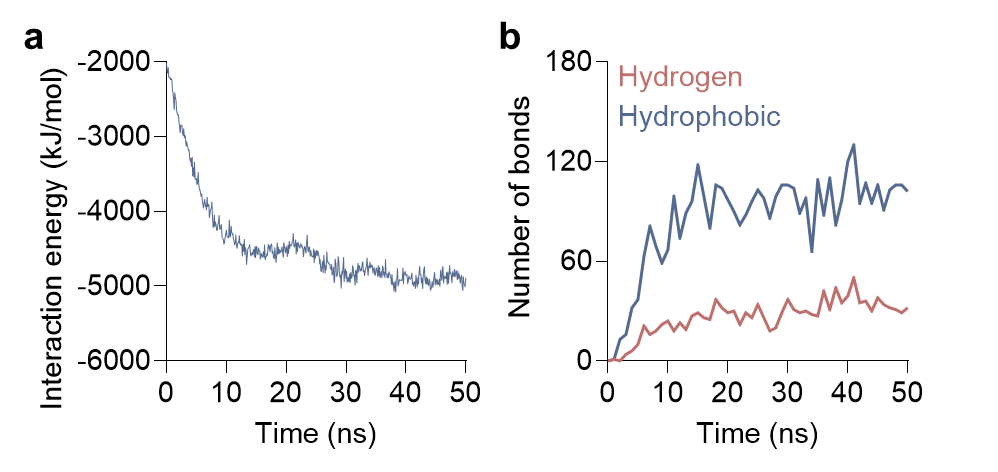
**

**Supplementary Figure 7. (a)** Time evolution of the total interaction energy among drug conjugate molecules during MD simulation. **(b)** Time course of the number of hydrogen bonds and hydrophobic interactions.


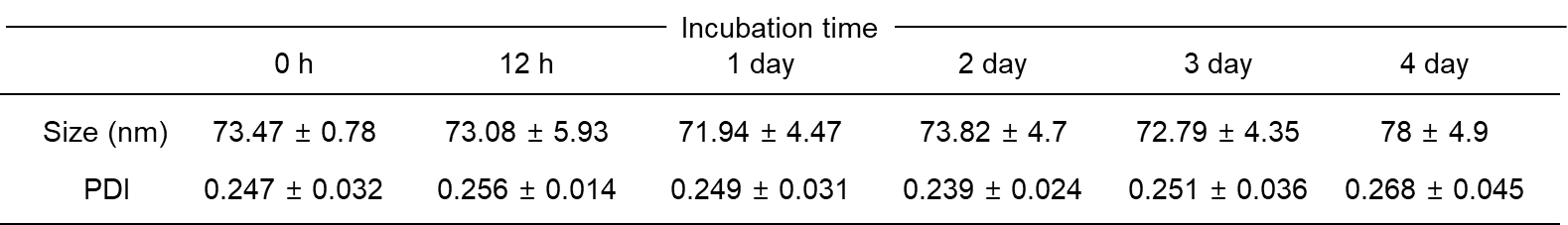


**Supplementary Figure 8.** Detailed information on the hydrodynamic size and polydispersity index (PDI) of NanoTAC during time-dependent incubation in mouse serum (n=5).


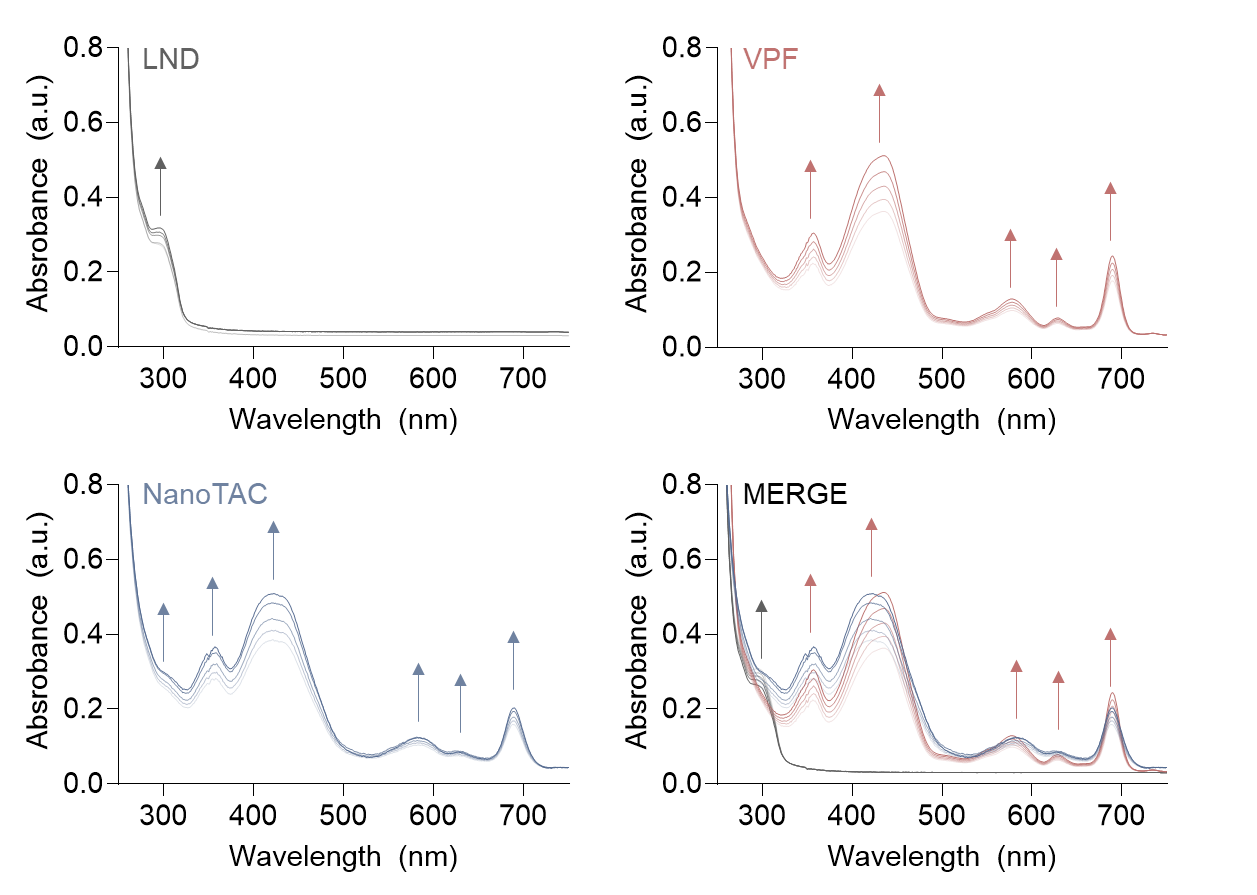


**Supplementary Figure 9.** UV-vis absorbance of lonidamine (LND), VPF and NanoTAC at varying concentrations.


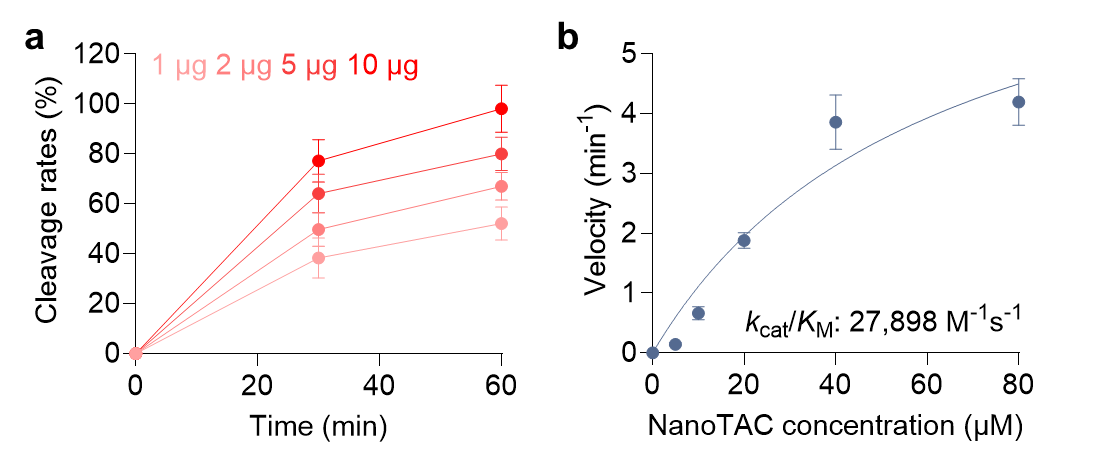


**Supplementary Figure 10. (a)** Cleavage behavior of NanoTAC after incubation with varying concentrations of Cat-B (1, 2, 5 and 10 μg). The cleavage rates (%) were calculated by comparing the peak area at each indicated time point to that of the same concentration of NanoTAC prior to incubation with Cat-B. **(b)** Catalytic efficiency (*k*_cat_/*K*_M_) determined from the reaction rates of the photosensitizer and PROTAC released after incubating varying concentrations of NanoTAC with Cat-B.


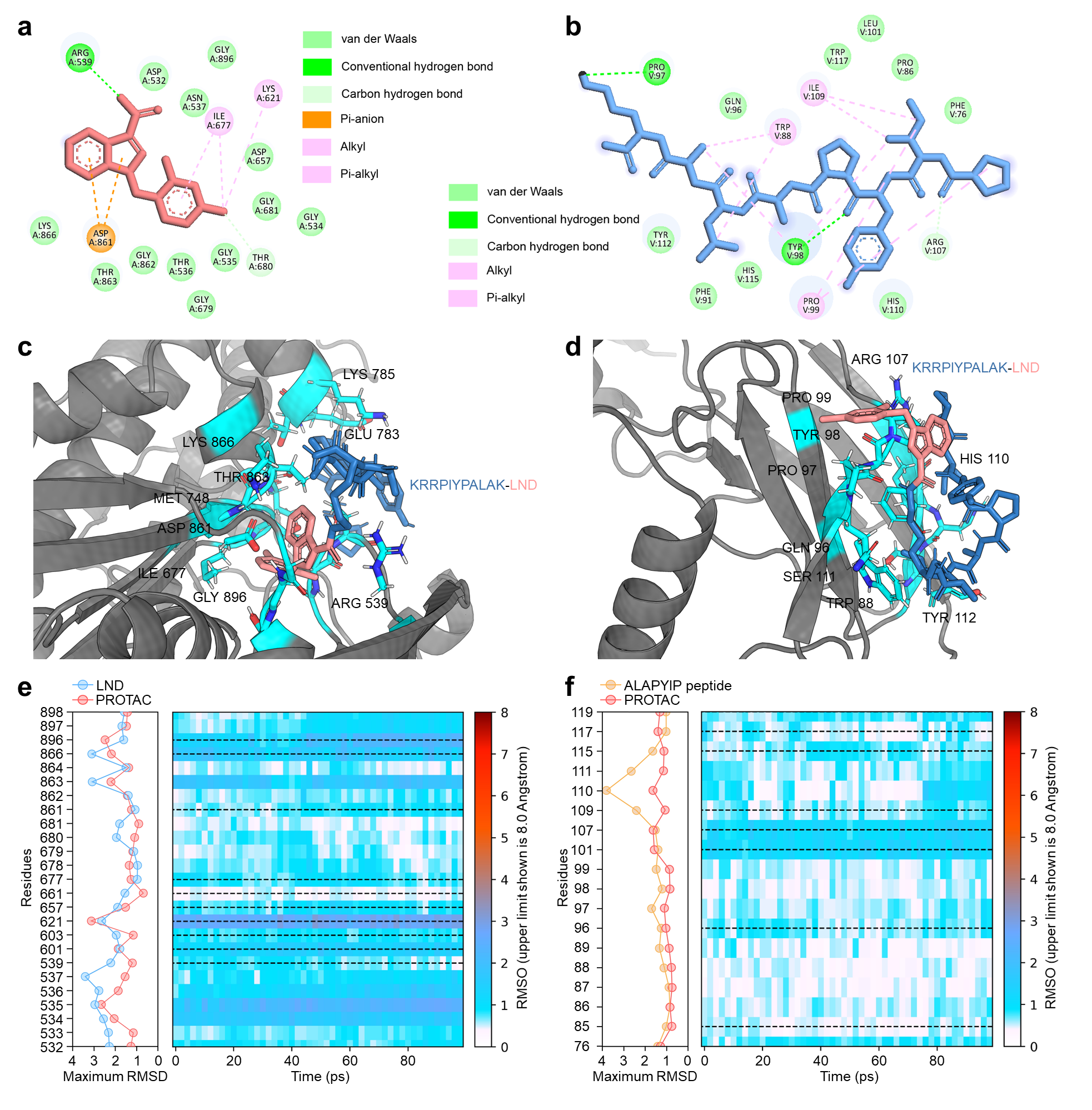


**Supplementary Figure 11.** Key non-covalent interaction types of **(a)** LND with HK2 (ASP861, ILE677, ARG539 and GLY896) and **(b)** ALAPYIP with VHL (ILE109, TRP88, TYR98, PRO99 and PRO97). Representative images showing successful docking poses of the PROTAC cleaved from NanoTAC with **(c)** HK2 and **(d)** VHL. Key interacting residues are indicated for each protein, and the PROTAC components of LND and the ALAPYIP peptide are represented in pink and blue, respectively. Root-mean-square deviation (RMSD) analysis comparing structural fluctuations of PROTAC-bound complexes to those bound to individual components (LND for HK2 and ALAPYIP peptide for VHL) during MD simulations. The PROTAC cleaved from NanoTAC exhibited reduced residue-level fluctuations at key interacting sites in the **(e)** HK2 and **(f)** VHL complexes compared to LND and ALAPYIP, respectively, indicating greater structural stabilization with each target protein.


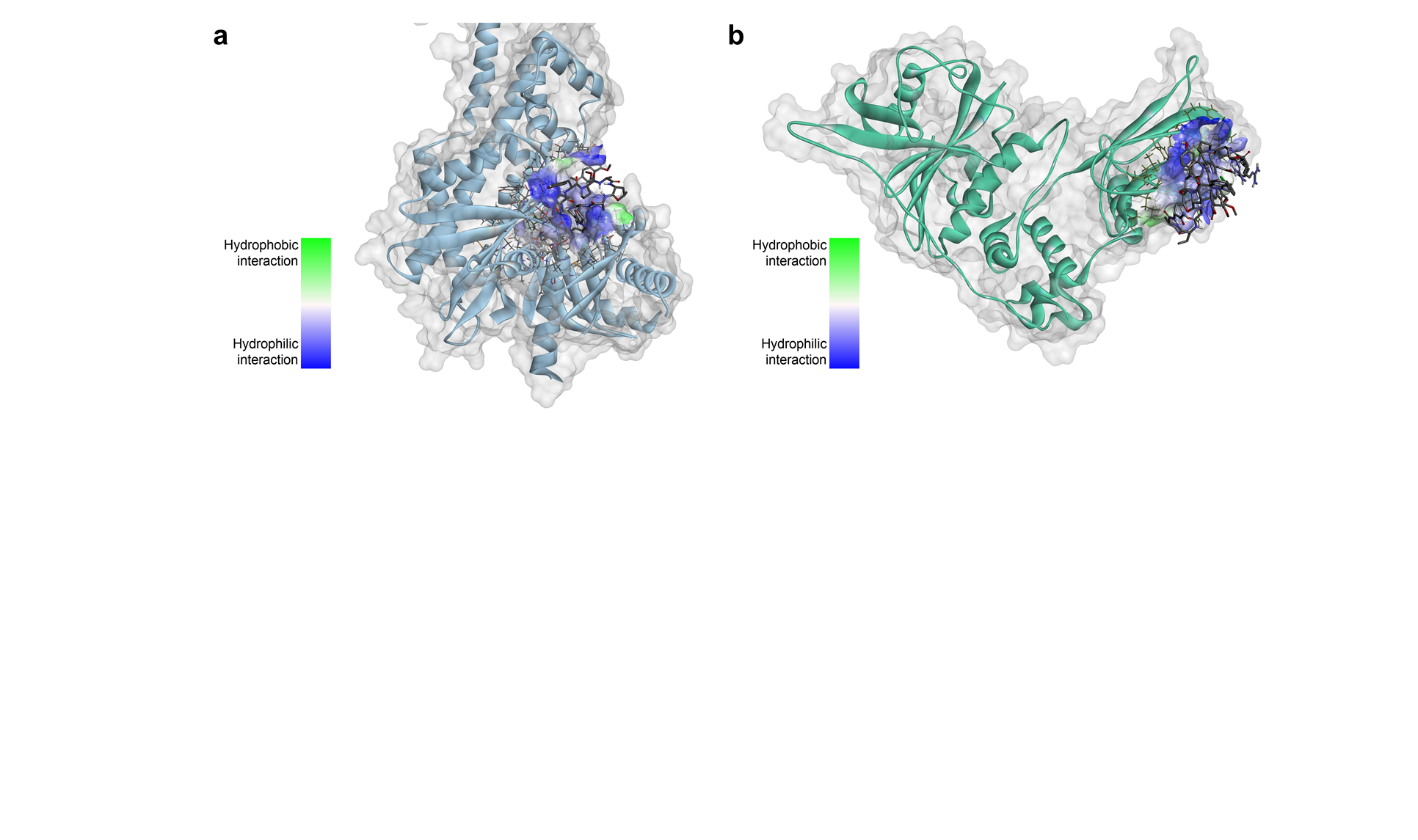


**Supplementary Figure 12.** Docking simulations of NanoTAC before enzymatic cleavage. Docking models of intact NanoTAC against **(a)** HK2 and **(b)** VHL proteins. Prior to cleavage, NanoTAC showed weak or no binding to either target, with affinities of 91.0 kcal/mol for HK2 and 8.0 kcal/mol for VHL, indicating unfavorable interactions.

**
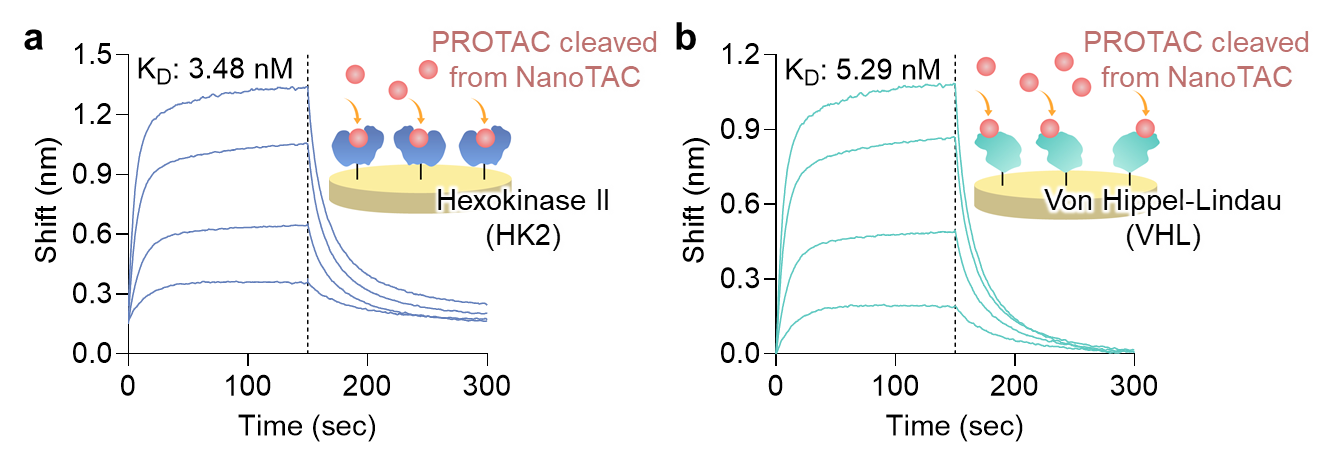
**

**Supplementary Figure 13.** Biolayer interferometry (BLI) analysis of the interactions between the PROTAC cleaved from NanoTAC and immobilized **(a)** HK2 and **(b)** VHL.


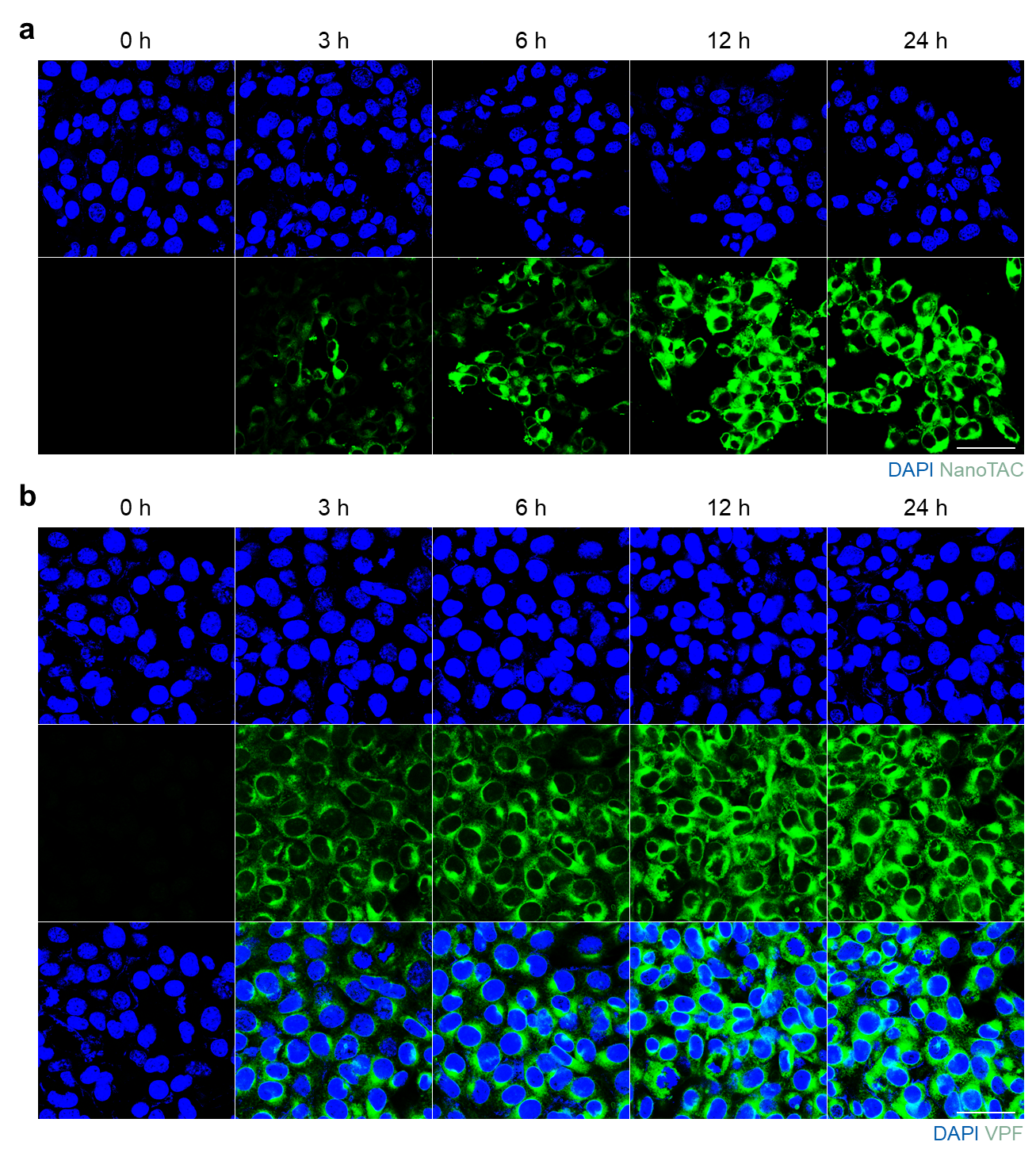


**Supplementary Figure 14.** Representative confocal microscopy images of 4T1 cells treated with **(a)** NanoTAC or **(b)** VPF for varying incubation times. Nuclei were counterstained with DAPI. Scale bar: 50 µm.


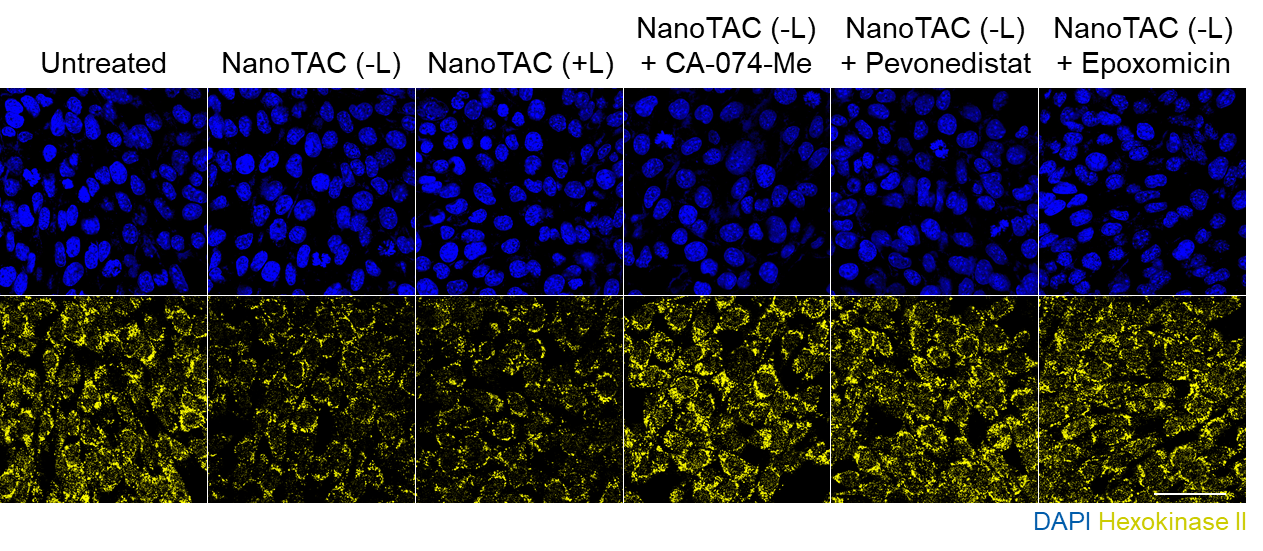


**Supplementary Figure 15. HK2 proteolysis by NanoTAC-mediated targeted protein degradation (TPD) in TNBC cells.** Representative confocal microscopy images of 4T1 cells treated with NanoTAC in the absence (-L) or presence (+L) of laser irradiation. As controls, 4T1 cells were pretreated with inhibitors targeting key steps in the TPD process, including the cathepsin B inhibitor CA-074-Me, the NEDD8-activating enzyme inhibitor pevonedistat and the proteasome inhibitor epoxomicin. HK2 and nuclei were counterstained with an anti-HK2 antibody and DAPI, respectively. Scale bar: 50 µm.


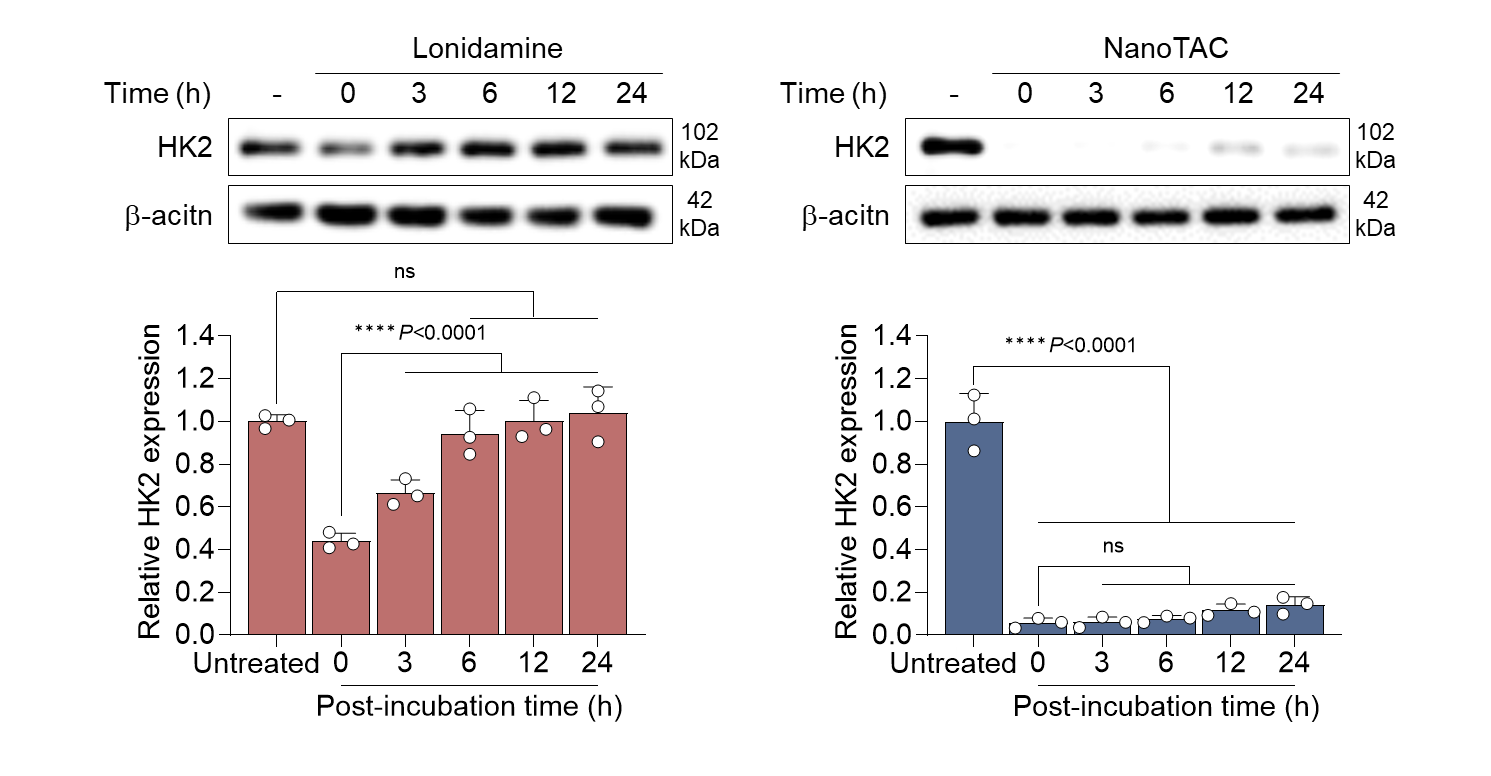


**Supplementary Figure 16.** Time-dependent HK2 expression in TNBC cells treated with 1 μM lonidamine or NanoTAC. The indicated time points (h) represent the duration after washing out lonidamine or NanoTAC following a 12 h treatment. Quantitative data are presented as mean ± SD (n=3). Statistical significance was determined by one-way ANOVA with Tukey-Kramer post-hoc test.


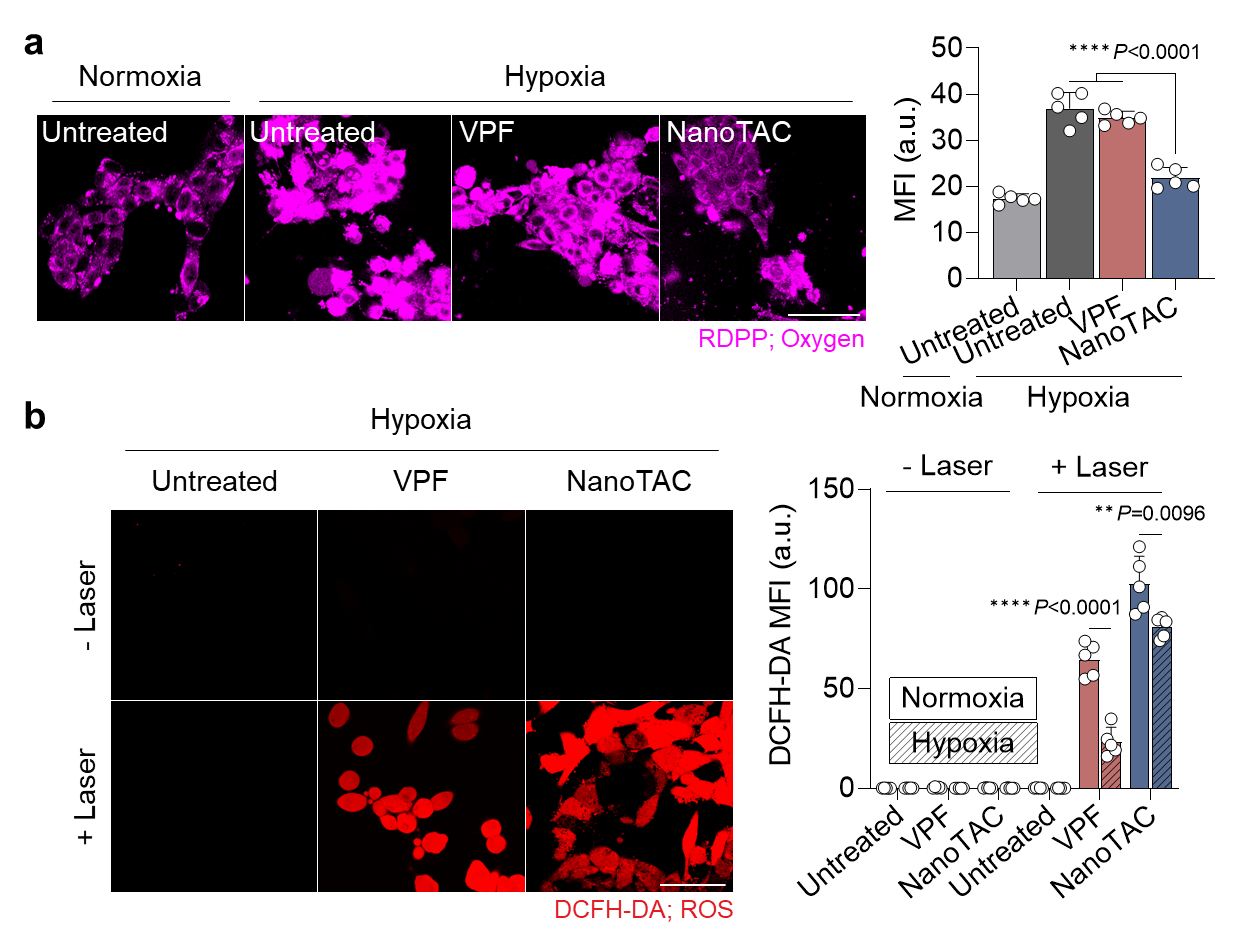


**Supplementary Figure 17. (a)** CLSM images showing oxygen levels in 4T1 cells under normoxic or hypoxic conditions after the indicated treatments (n=5). MFI data are presented as mean ± SD. Scale bar, 50 μm. **(b)** ROS generation in 4T1 cells under hypoxia following treatment with NanoTAC or VPF in the absence or presence of laser irradiation (40 mW, 500 s). MFI data are presented as mean ± SD (n=5). ROS generation under normoxia was quantified from the NIRF images shown in Figure 2c. Scale bar, 50 μm. Statistical significance was determined by one-way ANOVA with Tukey-Kramer post-hoc test.


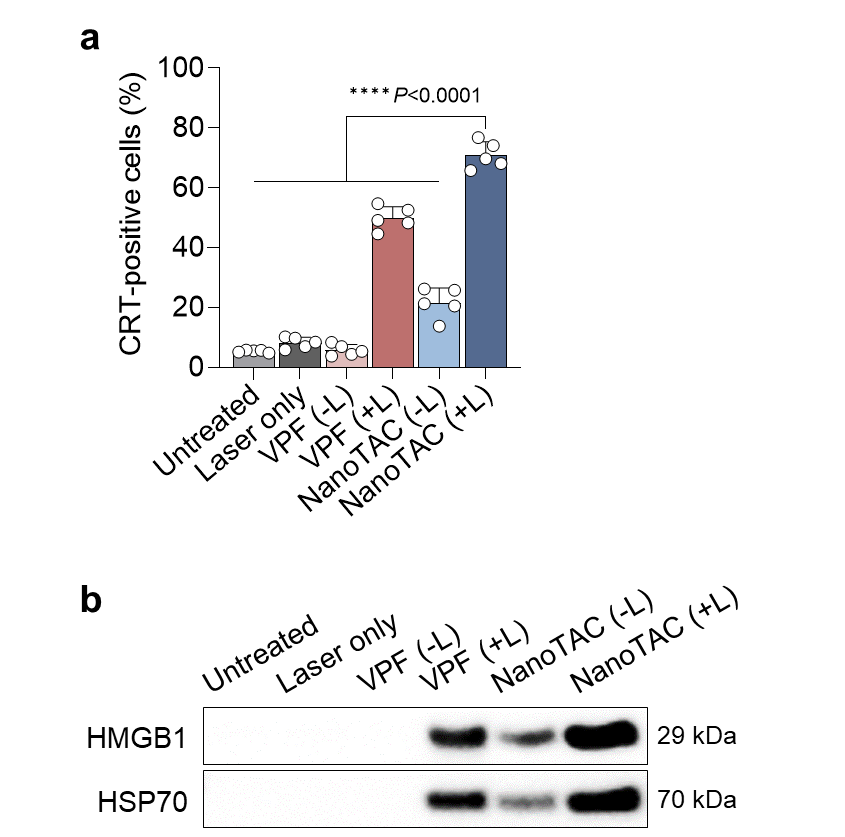


**Supplementary Figure 18. (a)** Percentage of surface calreticulin (CRT)-expressing 4T1 cells (gated as CD45^-^CRT^+^) following treatment with laser only, VPF plus laser (+L), NanoTAC without laser (-L) and NanoTAC (+L; n=5). **(b)** Representative western blot images showing levels of high mobility group box 1 (HMGB1) and heat shock protein 70 (HSP70) in the culture medium after treatment of 4T1 cells with laser only, VPF plus laser (+L), NanoTAC without laser (-L) and NanoTAC (+L). Statistical significance was determined by one-way ANOVA with Tukey-Kramer post-hoc test.


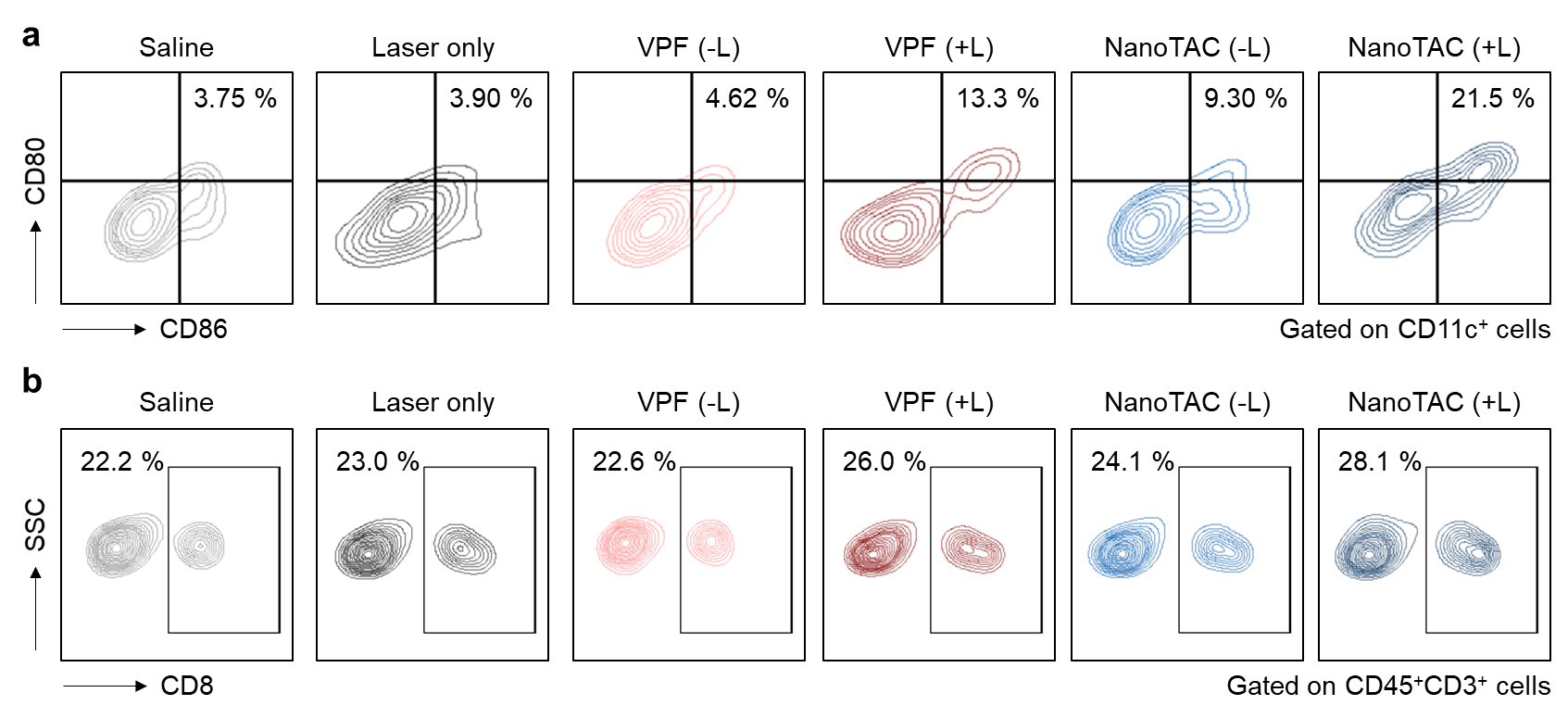


**Supplementary Figure 19.** Representative flow cytometry plots showing populations of **(a)** mature dendritic cells (DCs; CD11c^+^CD80^+^CD86^+^) and **(b)** cytotoxic T lymphocytes (CTLs; CD3^+^CD8^+^) in splenocytes after co-culture with 4T1 cells following treatment with laser only (Laser), VPF plus laser (+L), NanoTAC without laser (-L) and NanoTAC (+L).


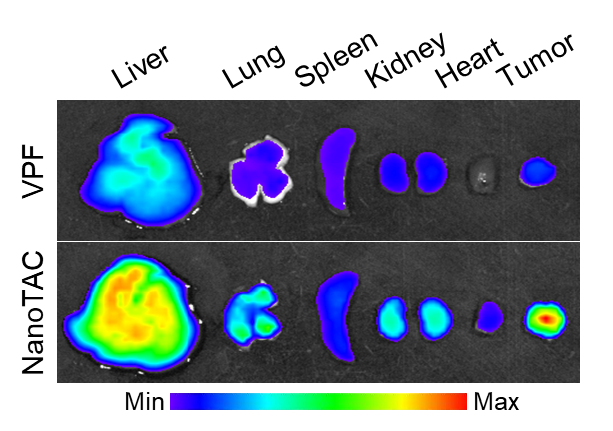


**Supplementary Figure 20.** Ex vivo NIRF images of major organs and tumor tissues resected from an orthotopic TNBC mouse model 3 h after intravenous administration of VPF or NanoTAC.


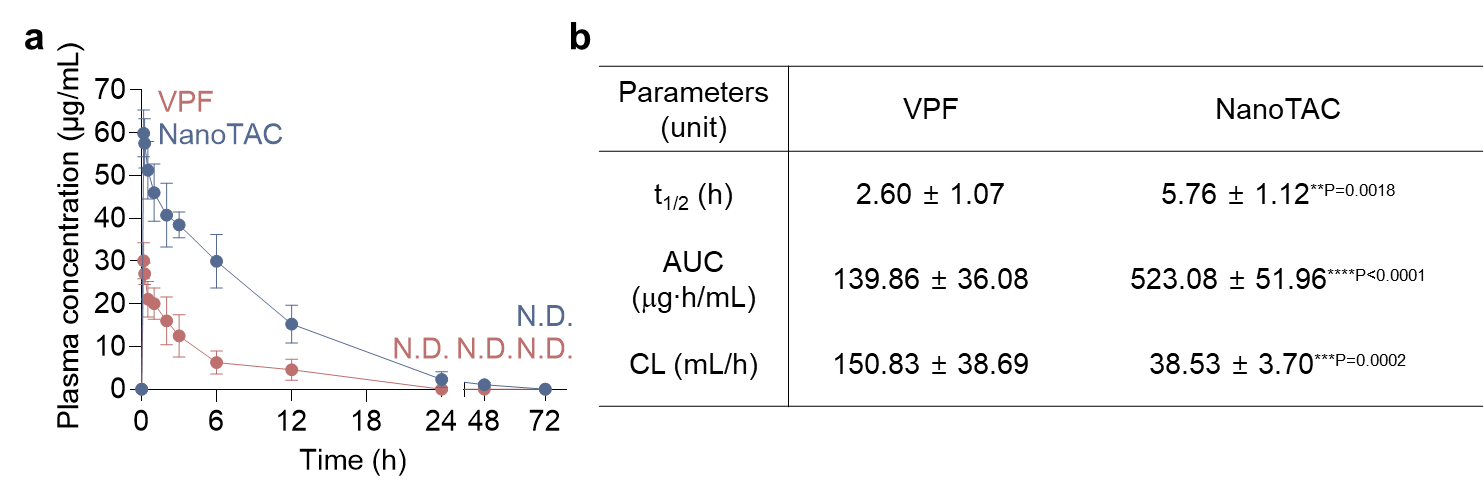


**Supplementary Figure 21. Pharmacokinetic study. (a)** Blood concentration-time profiles of NanoTAC and VPF following intravenous administration. **(b)** Pharmacokinetic parameters. Abbreviations: t_1/2_, half-life; AUC, Area under the curve from time 0 to the last sampling time; CL, total body clearance. N.D. indicates that NanoTAC (blue) or VPF (red) was not detected at the indicated time point. Data are presented as mean ± SD from five biologically independent mice per group (n=5). Statistical significance was determined by Student's t-test.

**
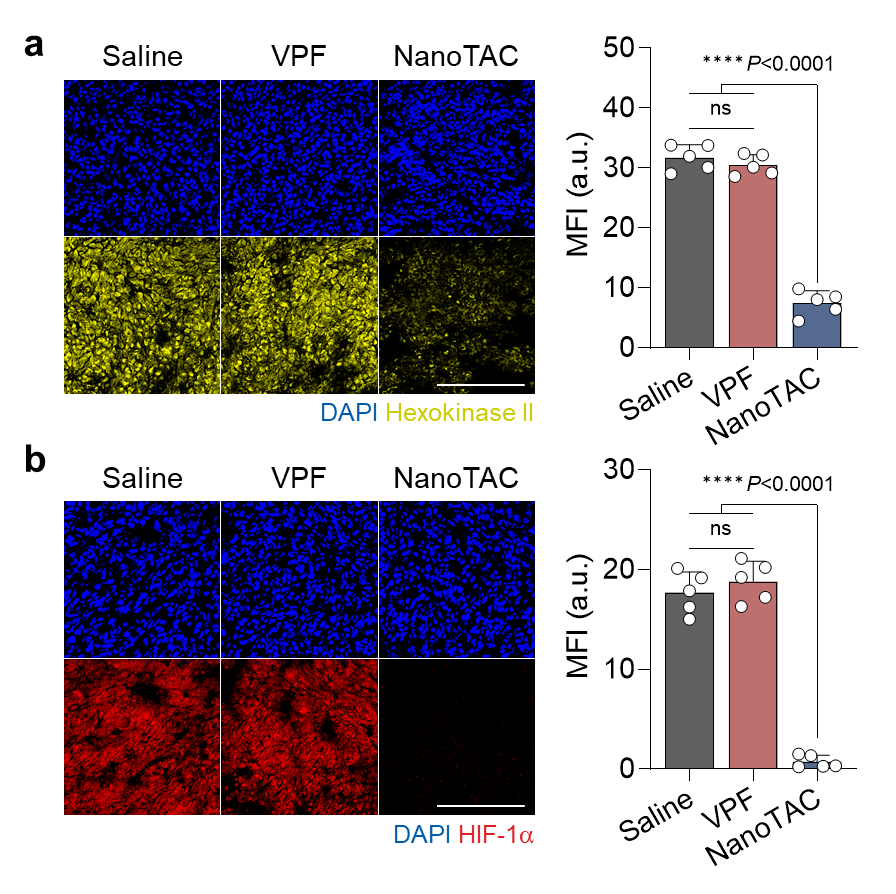
**

**Supplementary Figure 22.** Representative confocal microscopy images of tumor tissues stained for **(a)** HK2 or **(b)** HIF-1α. Scale bar, 100 μm. Quantitative data are presented as mean ± SD (n=5). Statistical significance was determined by one-way ANOVA with Tukey-Kramer post-hoc test.


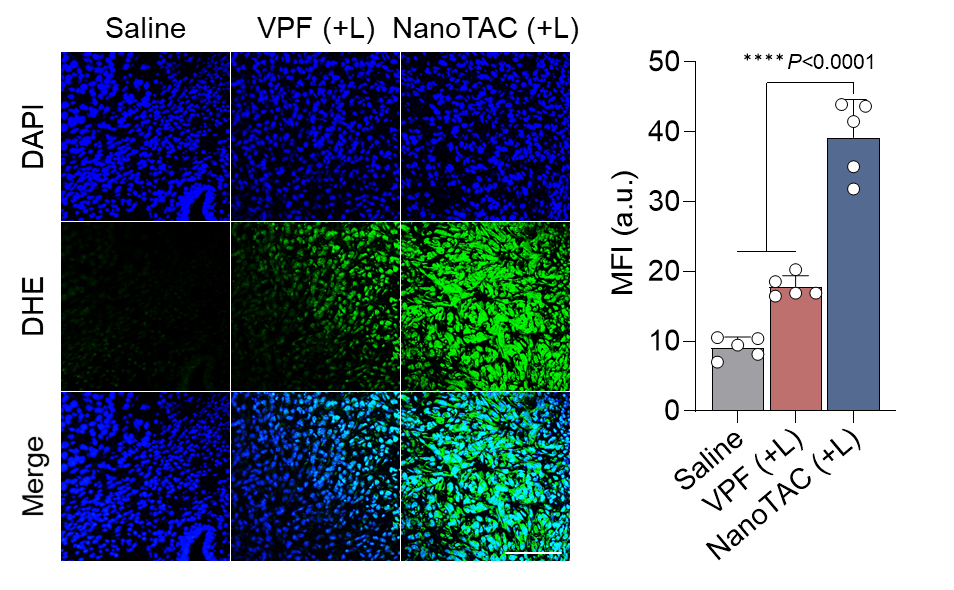


**Supplementary Figure 23.** Representative CLSM images of tumor tissues stained with DHE to visualize ROS. Scale bar, 100 μm. Quantitative data are presented as mean ± SD (n=5). Statistical significance was determined by one-way ANOVA with Tukey-Kramer post-hoc test.


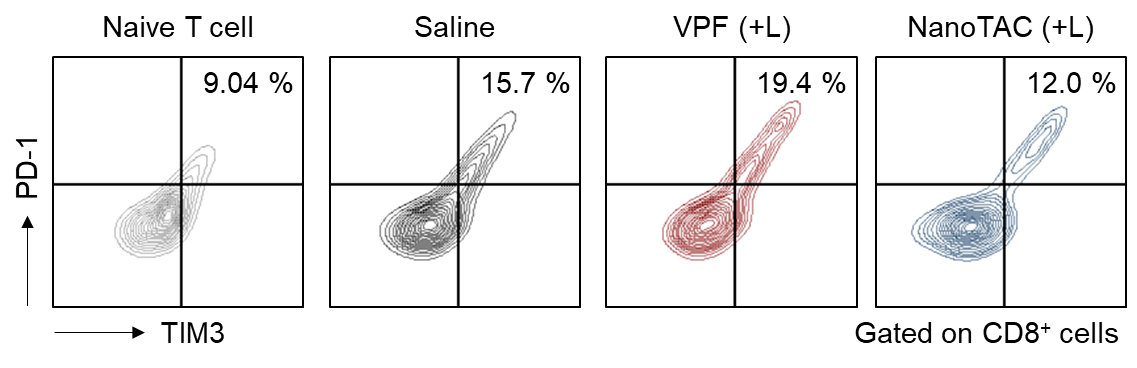


**Supplementary Figure 24.** Representative flow cytometry plots showing populations of T cells expressing the exhaustion markers PD-1 and TIM3 after incubation in tumor supernatants from TNBC mice treated with saline, VPF (+L) or NanoTAC (+L).

**Supplementary Figure 25.** Survival curves of TNBC mice in each group (n=5). Statistical significance was determined by log-rank test.

**Supplementary Figure 26.** Changes in body weight of orthotopic TNBC mice following treatment with Saline, laser only (Laser), VPF (+L) or NanoTAC (-L or +L; n=5). Statistical significance was determined by one-way ANOVA with Tukey-Kramer post-hoc test.


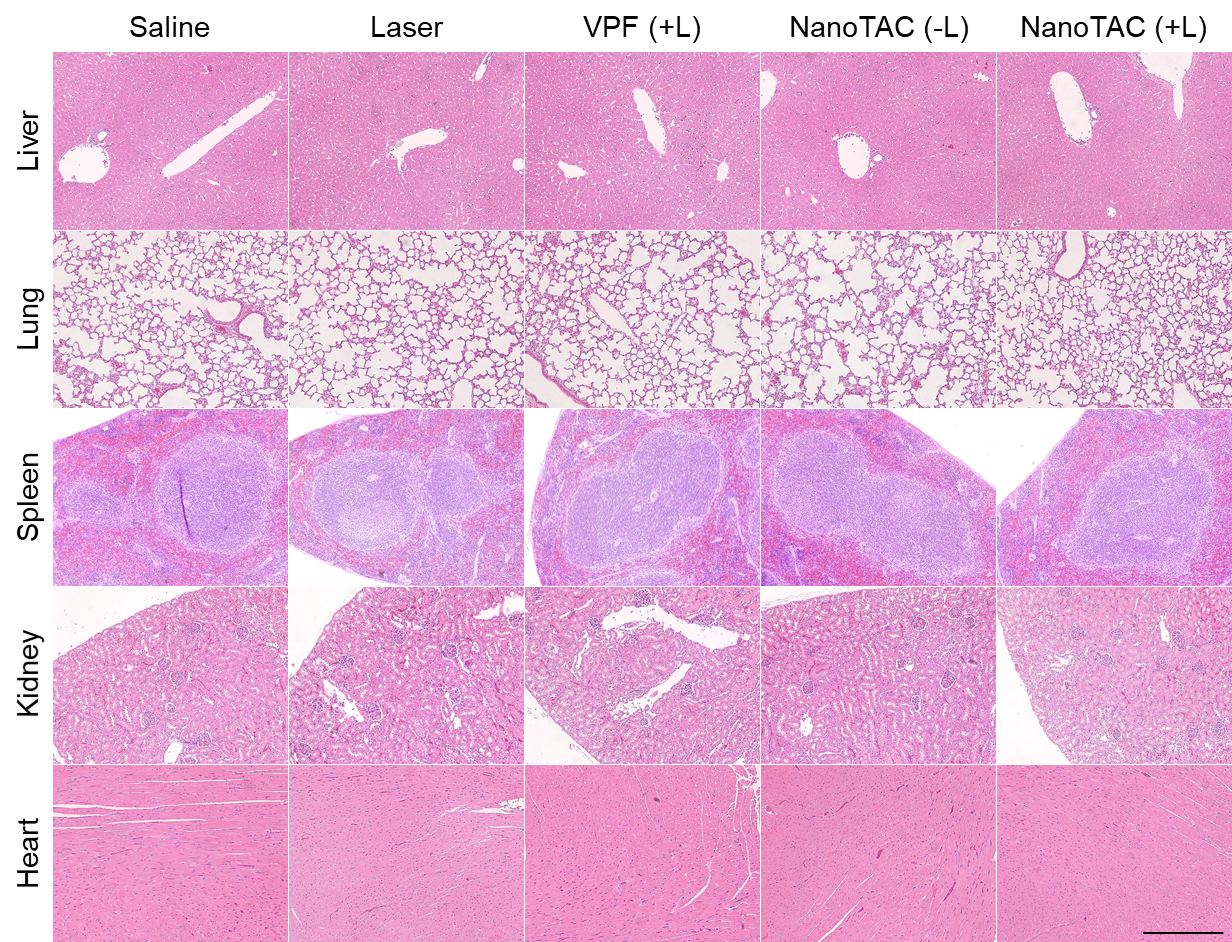


**Supplementary Figure 27.** Representative images of H&E-stained major organs collected from orthotopic TNBC mice following treatment with Saline, laser only (Laser), VPF (+L) or NanoTAC (-L or +L). VPF or NanoTAC was intravenously administered, followed by laser irradiation of the tumor tissues 3 h post-treatment. Scale bar: 200 µm.


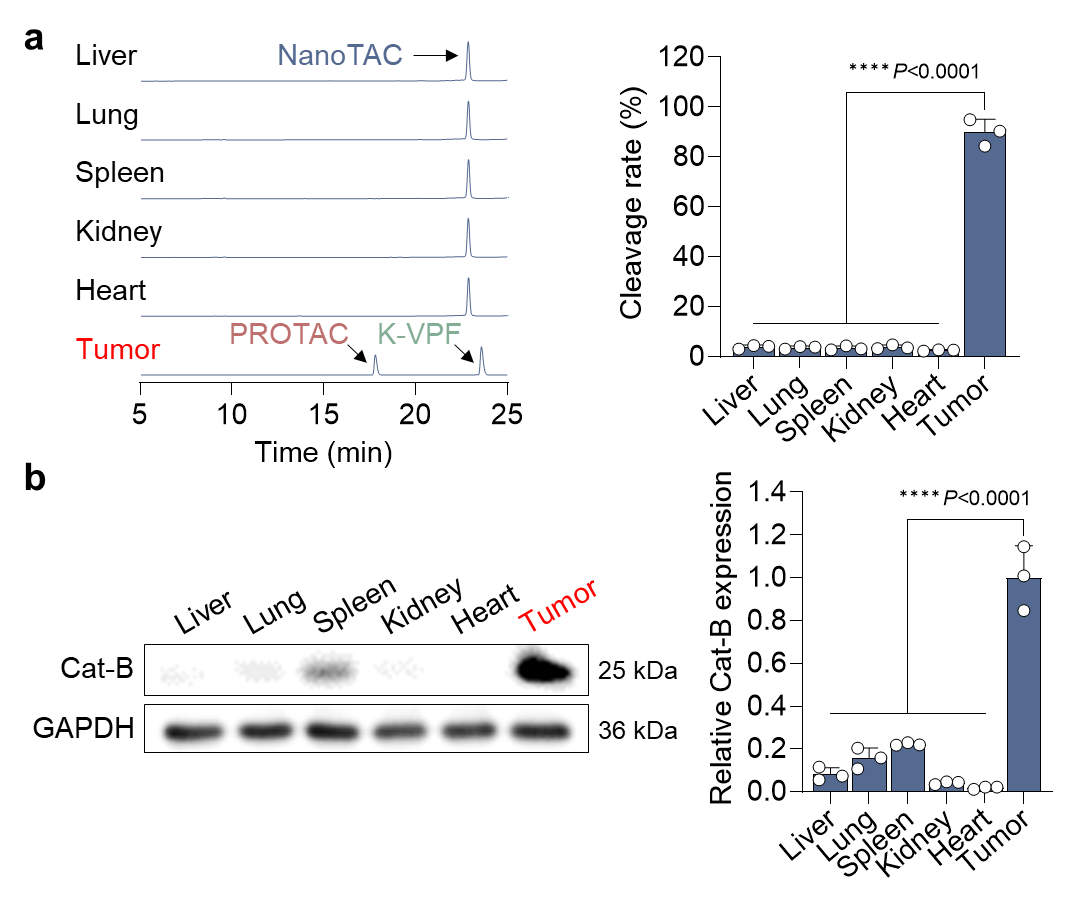


**Supplementary Figure 28. (a)** Cleavage behavior of NanoTAC after 24 h of incubation in lysates from individual organs and TNBC tissues. The cleavage rate (%) was calculated by comparing the peak area to that of an equivalent concentration of NanoTAC in distilled water. **(b)** Cathepsin B expression in normal organs and TNBC tissues. Quantitative data are presented as mean ± SD (n=3). Statistical significance was determined by one-way ANOVA with Tukey-Kramer post-hoc test.


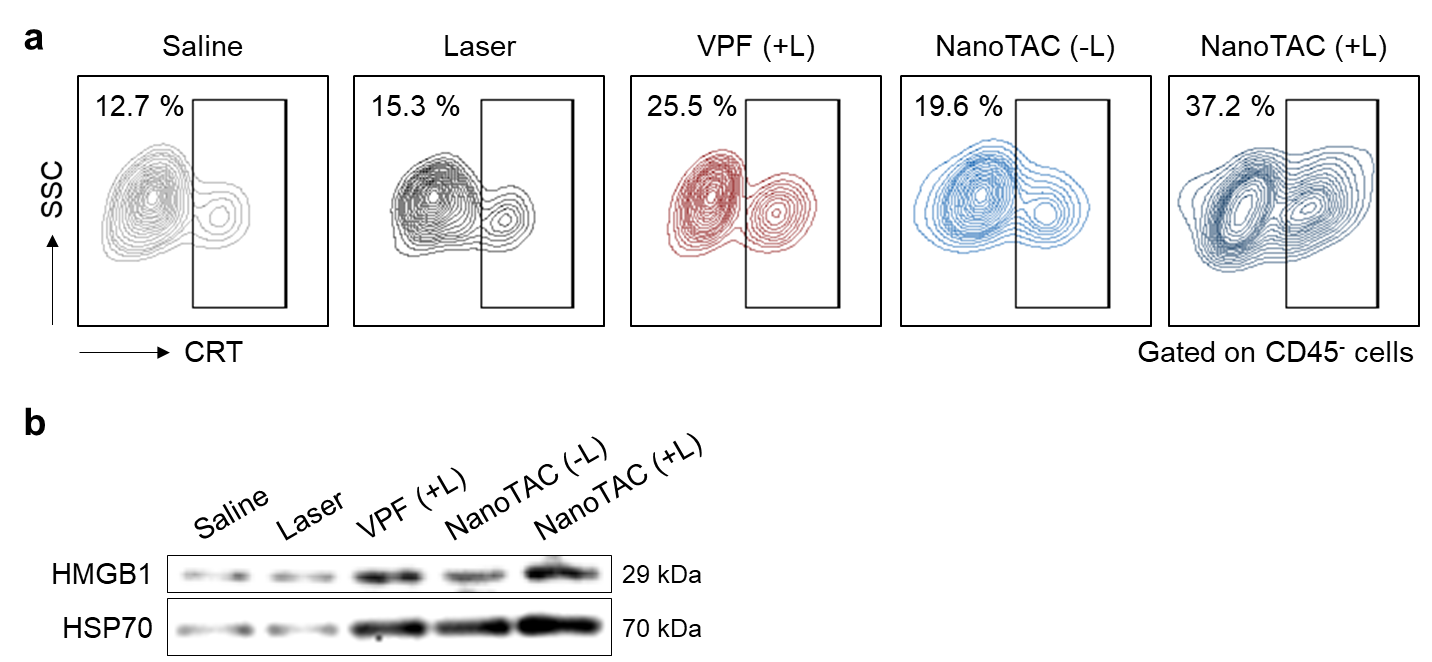


**Supplementary Figure 29. DAMP expression in TNBC tissues. (a)** Representative flow cytometry plots showing CRT-expressing tumor cells (CD45^-^CRT^+^) within tumor tissues from an orthotopic TNBC mouse model in each group. **(b)** Western blot images showing the extracellular release of HMGB1 and HSP70, analyzed in tumor supernatants.


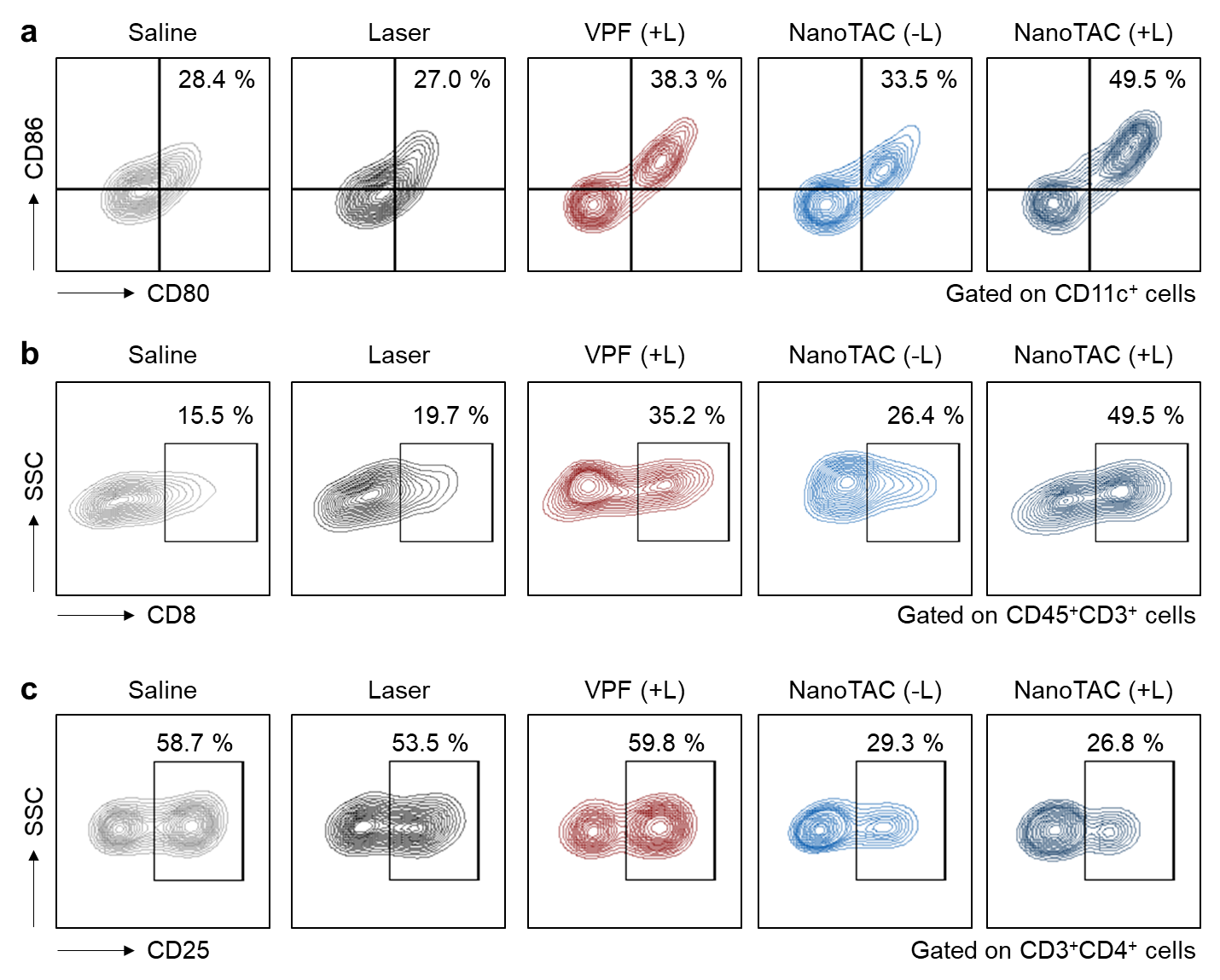


**Supplementary Figure 30.** Representative flow cytometry plots showing populations of **(a)** mature DCs (CD11c^+^CD80^+^CD86^+^), **(b)** CTLs (CD45^+^CD3^+^CD8^+^) and **(c)** regulatory T (T_reg_; CD3^+^CD4^+^CD25^+^) cells within tumor tissues from an orthotopic TNBC mouse model in each group.


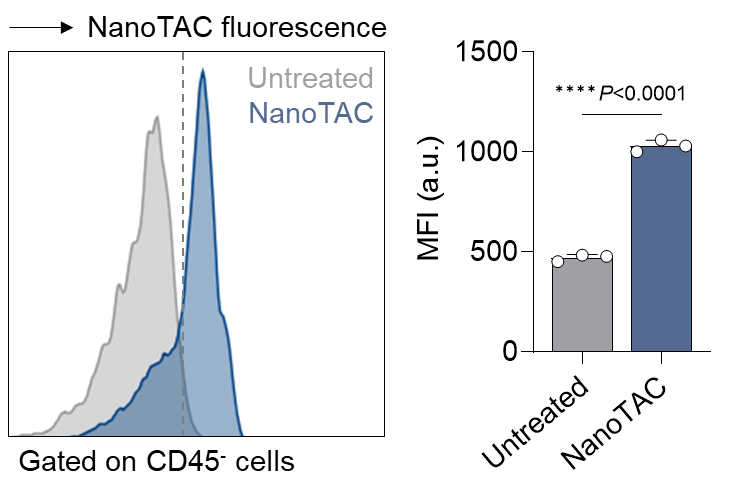


**Supplementary Figure 31. Quantification of NanoTAC in tumor cells administered to induce pulmonary metastasis.** After systemic administration of NanoTAC in orthotopic TNBC mice and subsequent laser irradiation 3 h post-treatment, 4T1 cells were injected intravenously. NanoTAC fluorescence in tumor cells gated as CD45-negative in blood was quantified by flow cytometry. Quantitative data are presented as mean ± SD (n=3). Statistical significance was determined by Student's t-test.

**
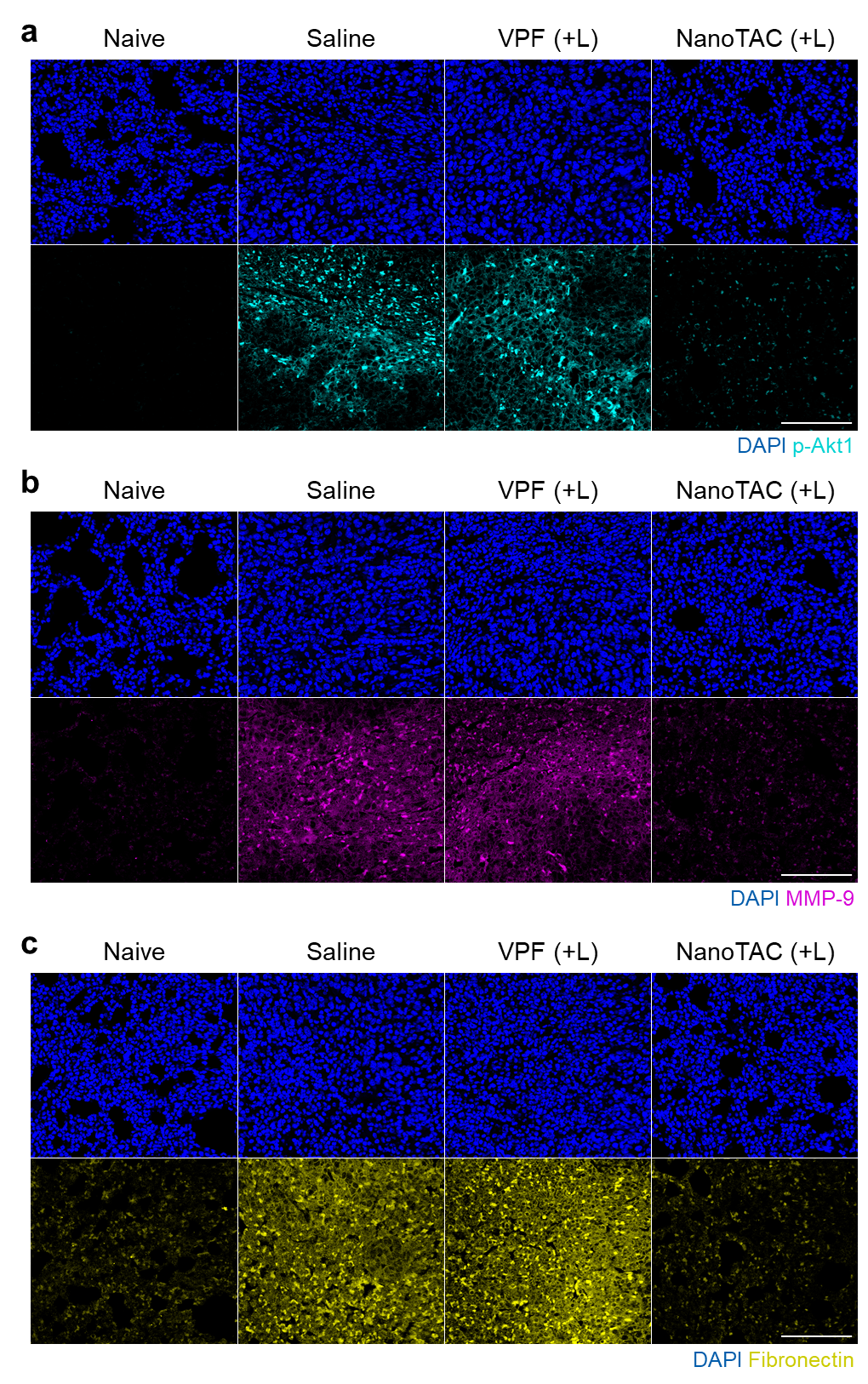
**

**Supplementary Figure 32.** Representative confocal microscopy images of pulmonary metastatic TNBC tissues from the naive group or mice treated with saline, VPF (+L) or NanoTAC (+L). Nuclei, along with **(a)** p-Akt1, **(b)** MMP-9 or **(c)** fibronectin, were counterstained using DAPI and antibodies against each respective protein. Scale bar: 50 µm.

**
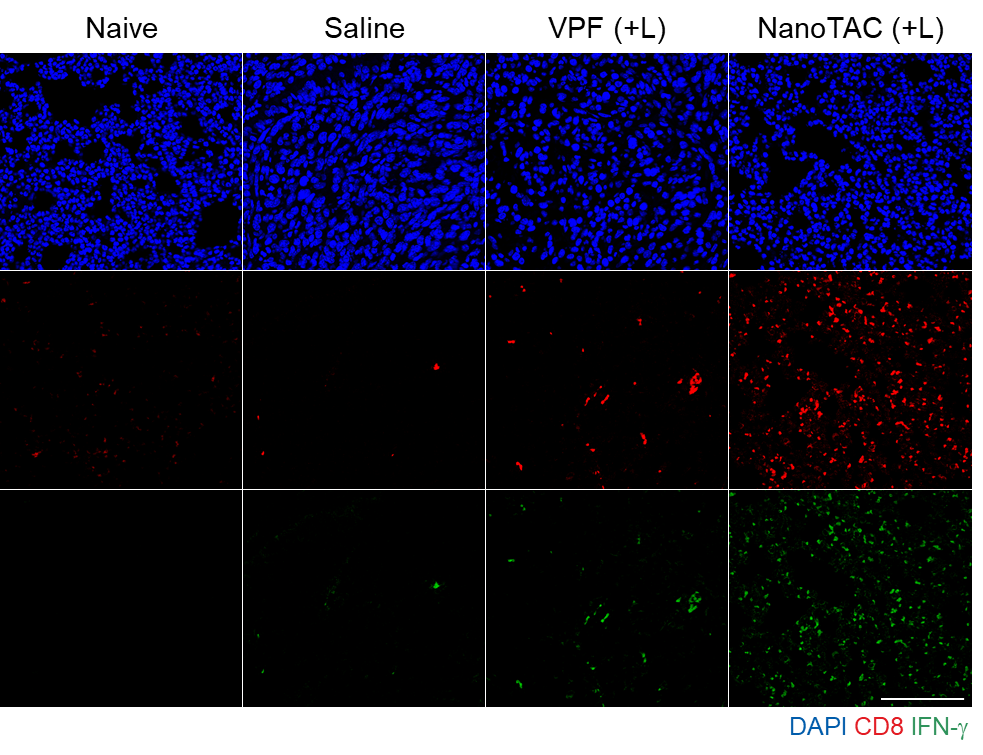
**

**Supplementary Figure 33.** Representative confocal microscopy images of pulmonary metastatic TNBC tissues from the naive group or mice treated with saline, VPF (+L) or NanoTAC (+L). Nuclei, along with CD8 and IFN-γ, were counterstained using DAPI and antibodies against each respective protein. Scale bar: 50 µm.
