# Supplementary figures and images for: Reprogramming of cancer metabolism via photoresponsive nano-PROTAC enhances pyroptosis-mediated immunotherapy
Source: Signal Transduct Target Ther. 2025 Sep 26;10:310. doi: 10.1038/s41392-025-02405-6 (PMC12464334; doi:10.1038/s41392-025-02405-6)

**Fig. 3a**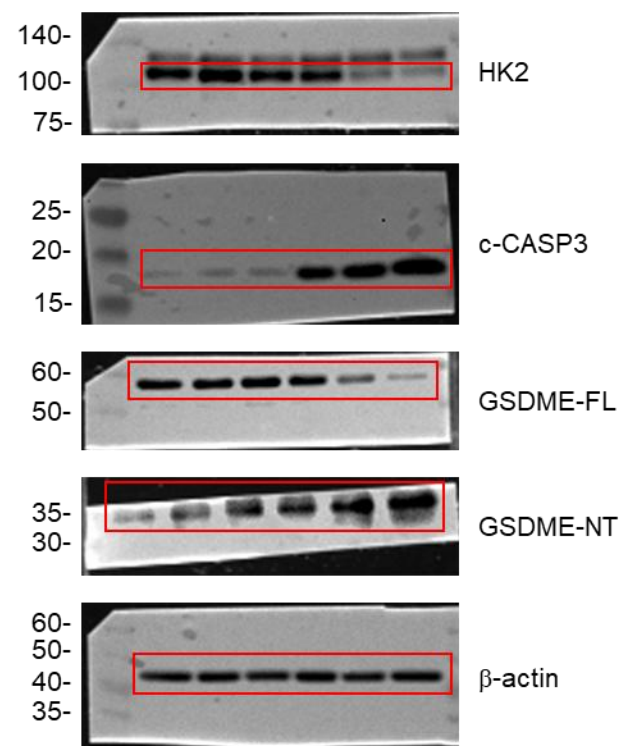**Fig. 6b**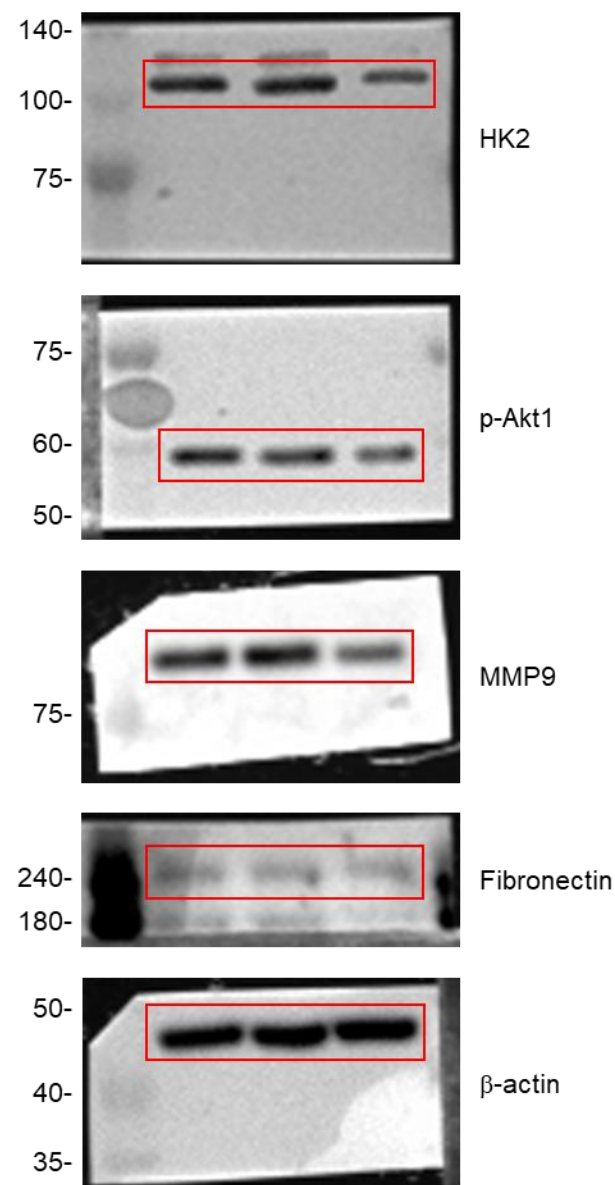**Fig. S16**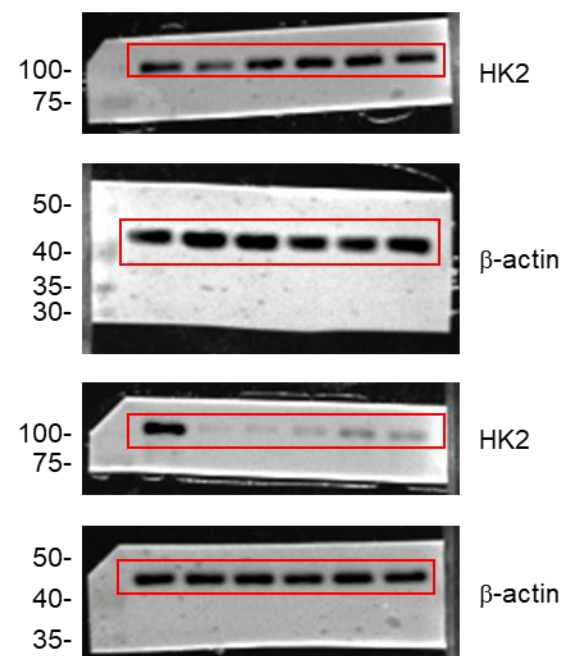**Fig. S18b**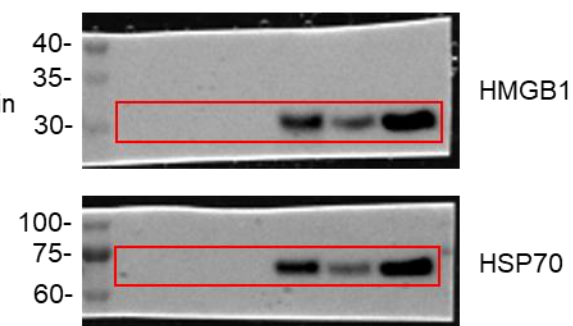**Fig. S28b**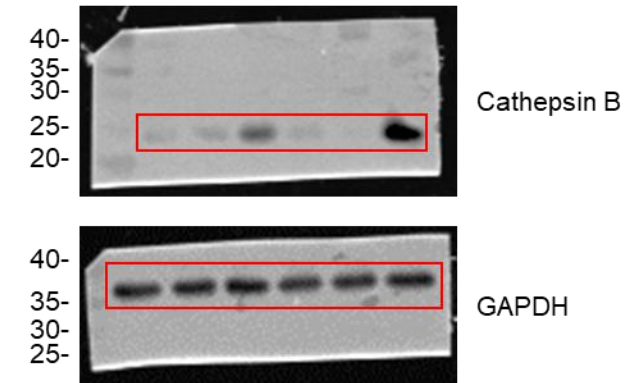**Fig. S29b**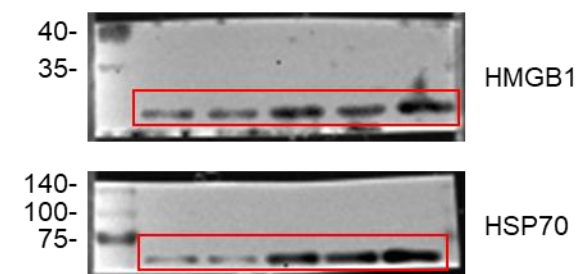

Supplement: Supplementary file 2 — Uncropped Western Blot Images [file 41392_2025_2405_MOESM2_ESM.pdf]
